# Supplementary material for: Effect of imputation on gene network reconstruction from single-cell RNA-seq data
Source: Patterns (N Y). 2021 Dec 22;3(2):100414. doi: 10.1016/j.patter.2021.100414 (PMC8848013; doi:10.1016/j.patter.2021.100414)
Supplement: Document S1. Figures S1–S7 and Tables S1 and S2 [file mmc1.pdf]

Patterns, Volume *3*

Supplemental information

Effect of imputation on gene network reconstruction from single-cell RNA-seq data

Lam-Ha Ly and Martin Vingron

Supplemental Information

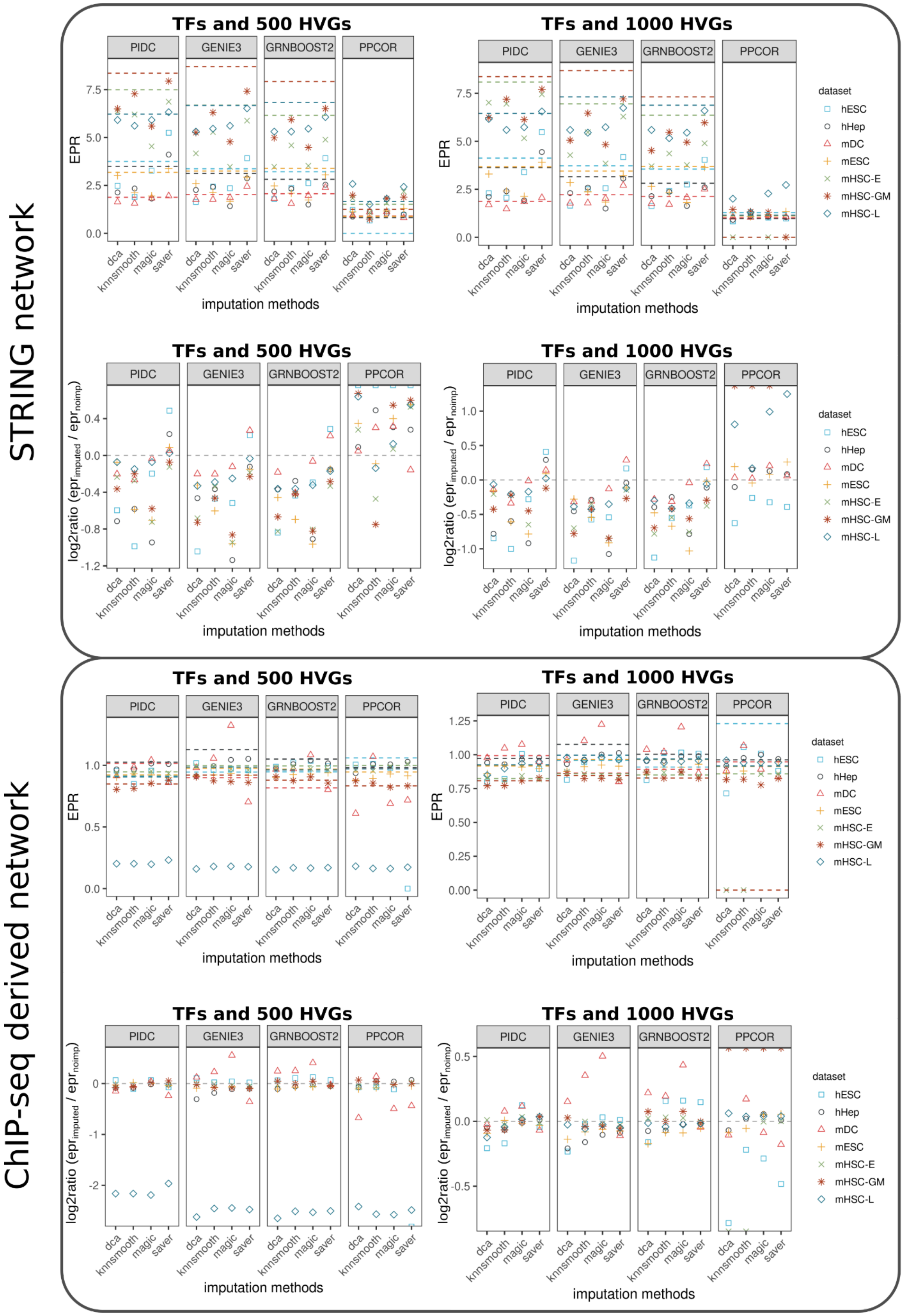

Fig. S1 | Performance scores of network models including PPCOR compared to STRING and ChIP-seq derived networks as evaluation networks. Upper panel is related to to Fig. 2 comparing

the inferred network results with the STRING network. Here, we include PPCOR as a baseline GRN algorithm. PPCOR performs almost similarly to a random predictor. In some cases PPCOR failed to run due to ill-conditioned data matrices corresponding to EPR scores equal to 0. Below panel compares the inferred network results with cell type-specific ChIP-seq derived networks. In both prefiltered datasets the performances are close to random. Imputation does not improve the network predictions. Due to normalization by using the network density, the EPR scores in mHSC-L imputed data differ strongly from the unimputed data. Here, low numbers of genes/ TFs and edges lead to different network densities (see Data S1). In both evaluation scenarios the results between the prefiltered datasets based on the number of highly variable genes (HVGs) are comparable. Hence, varying the number of genes has little effect on the network performance predictions.

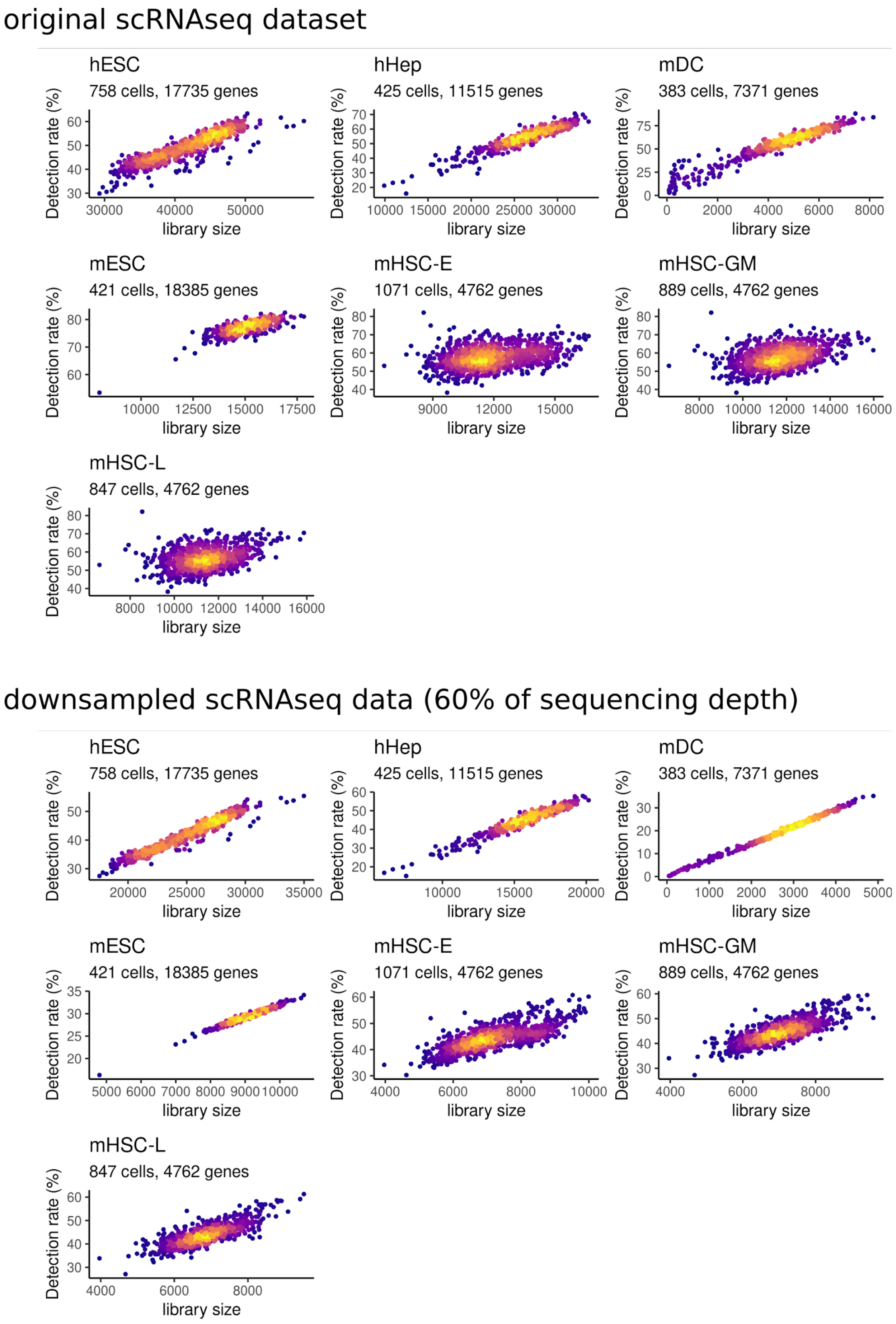

Fig. S2 | Gene detection rate and library size in experimental scRNAseq datasets (original and downsampled). Scatterplots colored by density of points (cells). Gene detection using a threshold of gene count > 0. Library size determined by the sum of all gene counts. Downsampling procedure

performed by sampling n times (60% of the original library size) according to the multinomial distribution.

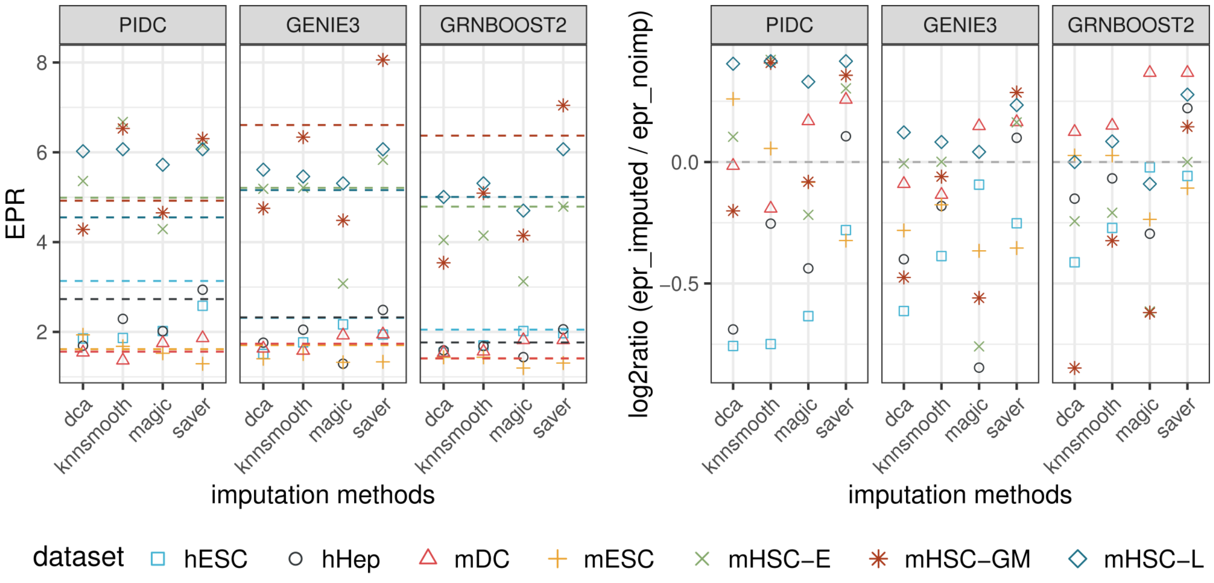

Fig. S3 | Performance measures of network models obtained by downsampled dataset. Performance scores reported on downsampled scRNAseq data (60% of original library size) and prefiltered dataset (TFs and 500 HVGs). (Left) Absolute EPR scores. Dashed line represents EPR scores obtained without imputation. (Right) log2-ratios between imputed and unimputed EPR scores. Log2ratio = 0 represents no change in performance (grey dashed line) after imputation. Generally, more improvements (positive log2ratios) than in the respective column of Figure 2 (TFs and 500 HVGs).

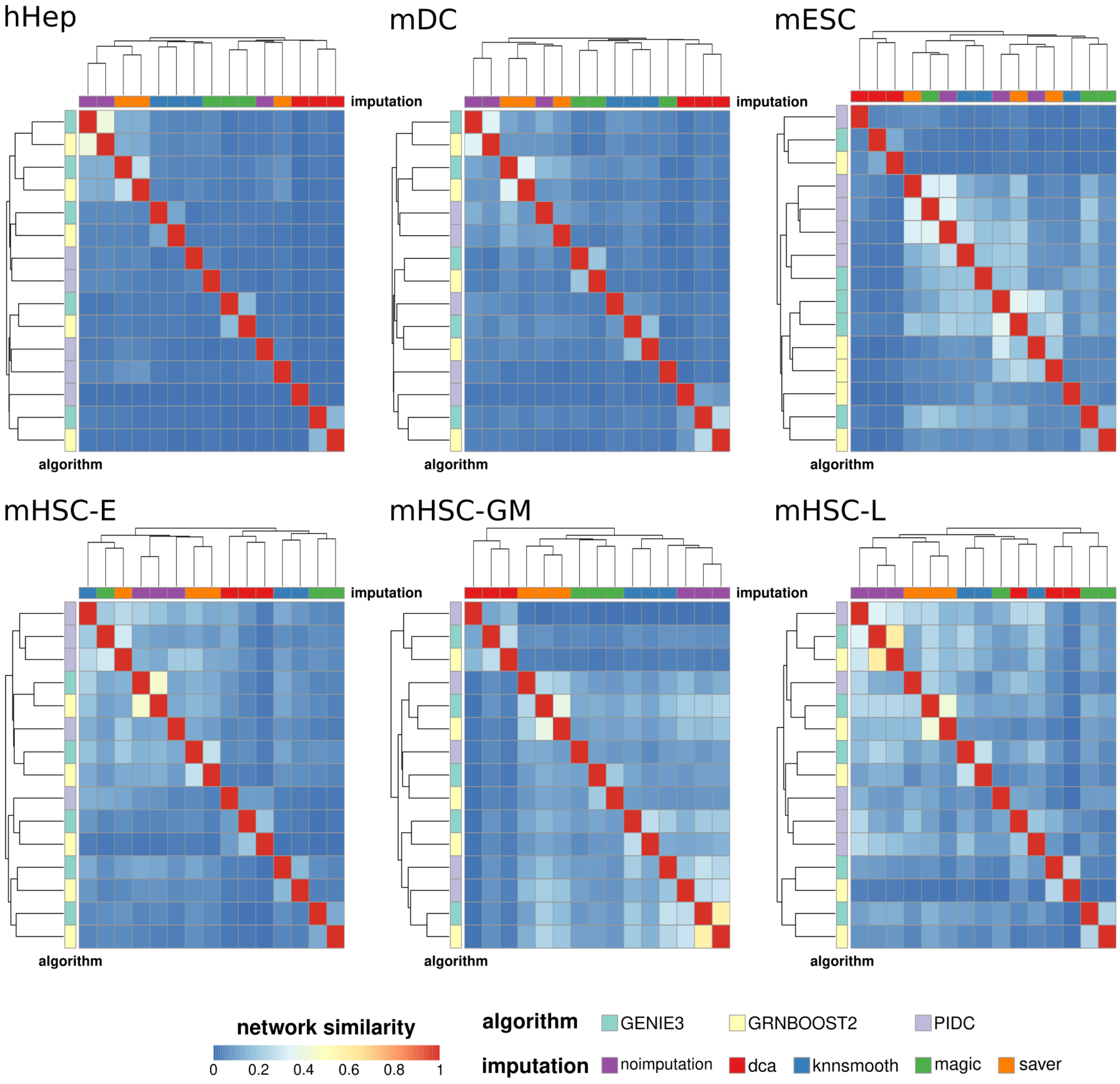

Fig. S4 | Network similarities across all models and cell types. Related to Figure 3B we inspect the heatmap of network similarities of the remaining cell types. Network similarity scores obtained by pairwise Jaccard index from top500 interactions. Columns are annotated by imputation method, rows are annotated by GRN reconstruction algorithm.

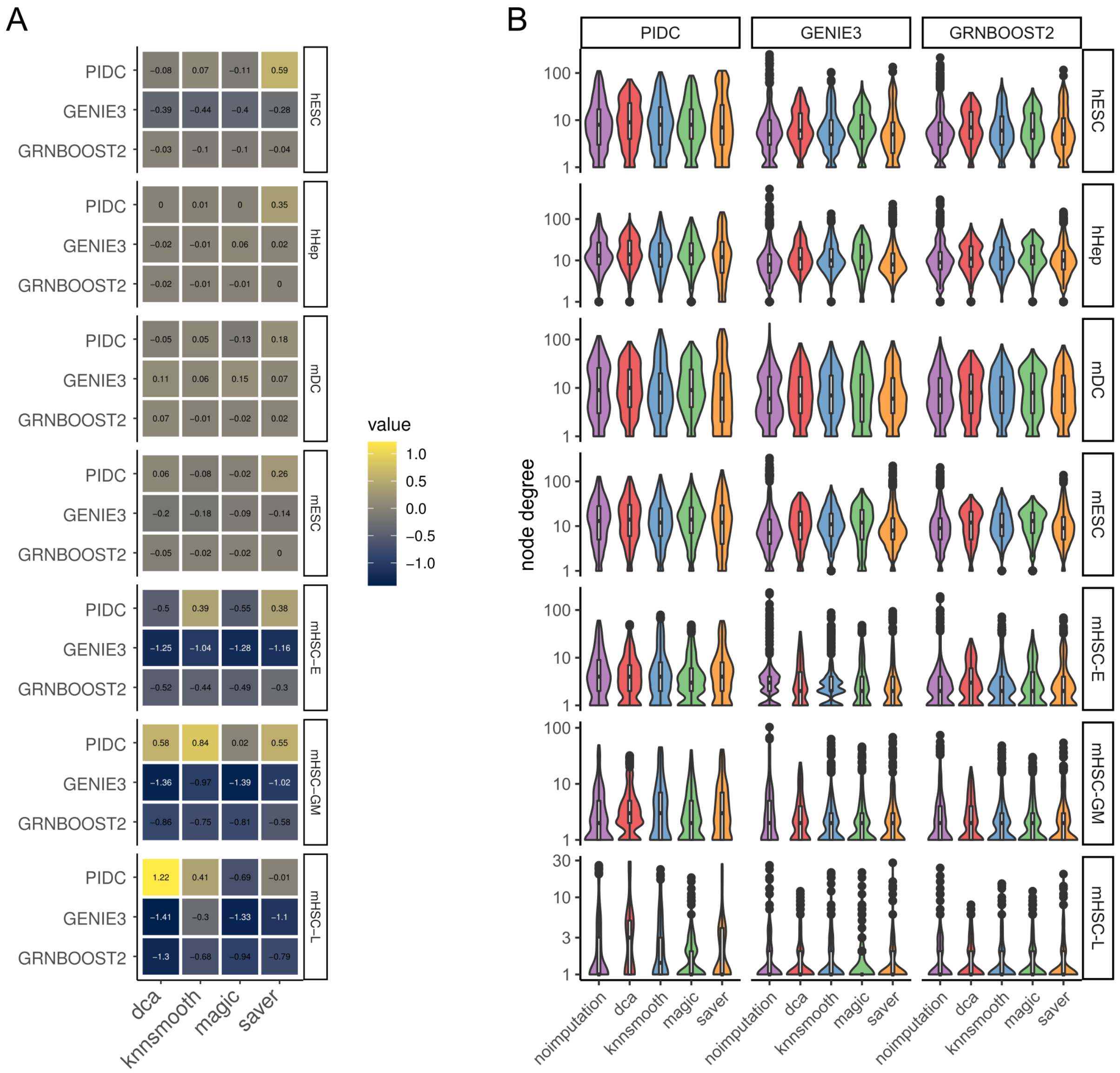

Fig. S5 | Structural changes in inferred networks. (A) Change of node density before and after imputation. Log2 ratios between density (after imputation) and density (before imputation) are color-coded. Positive values represent a denser whereas negative values represent a more sparse network with respect to the unimputed model. (B) Node degree distribution across all models. Y-axis is log-scaled.

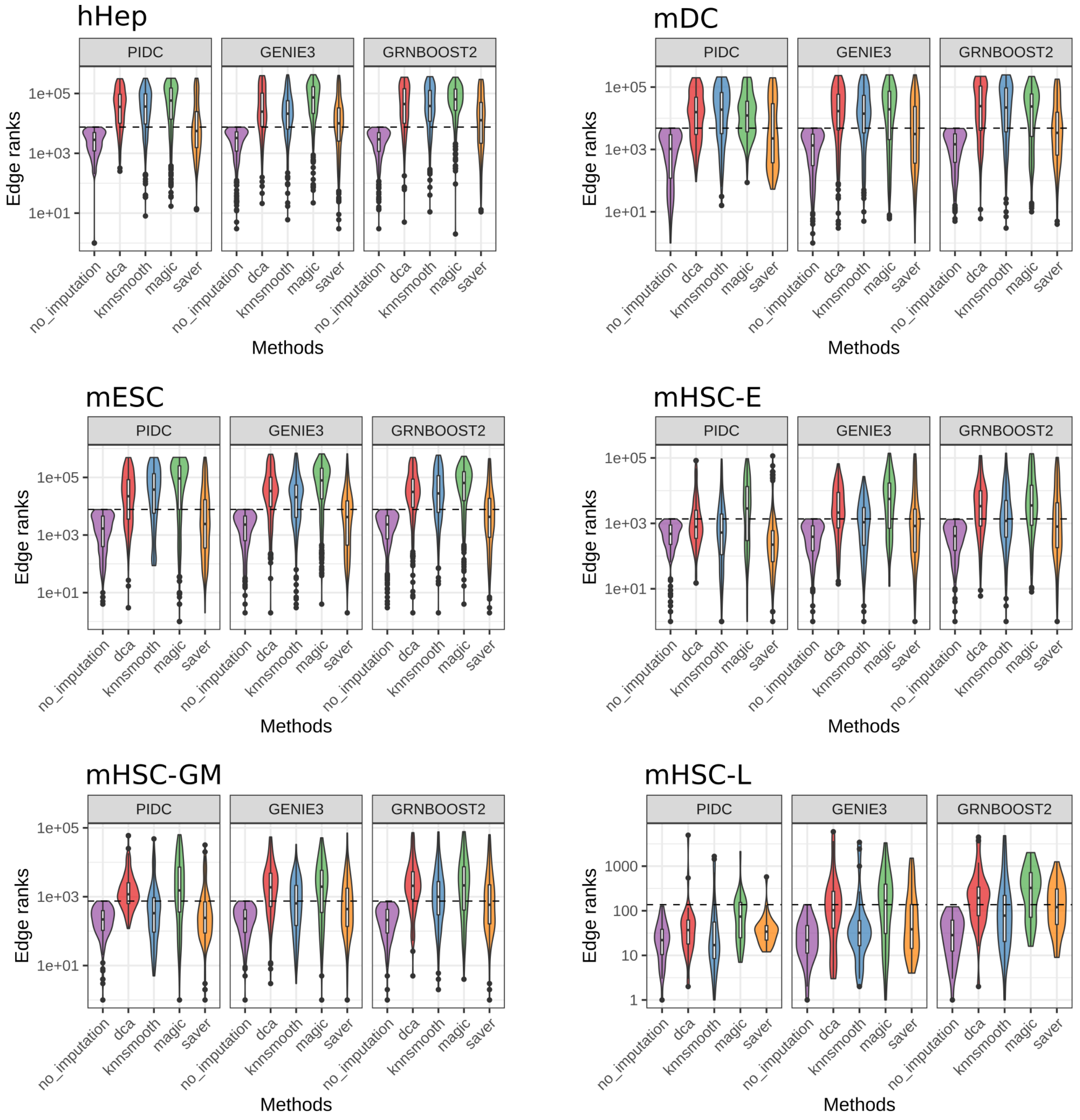

Fig. S6: True positive interactions identified on unimputed data and their change in edge ranks after imputation. Related to Figure 4C we inspect the change of unimputed TP ranks after imputation in the remaining cell types. Corrected p-values obtained by Wilcoxon rank sum test can be taken from Tab. S2.

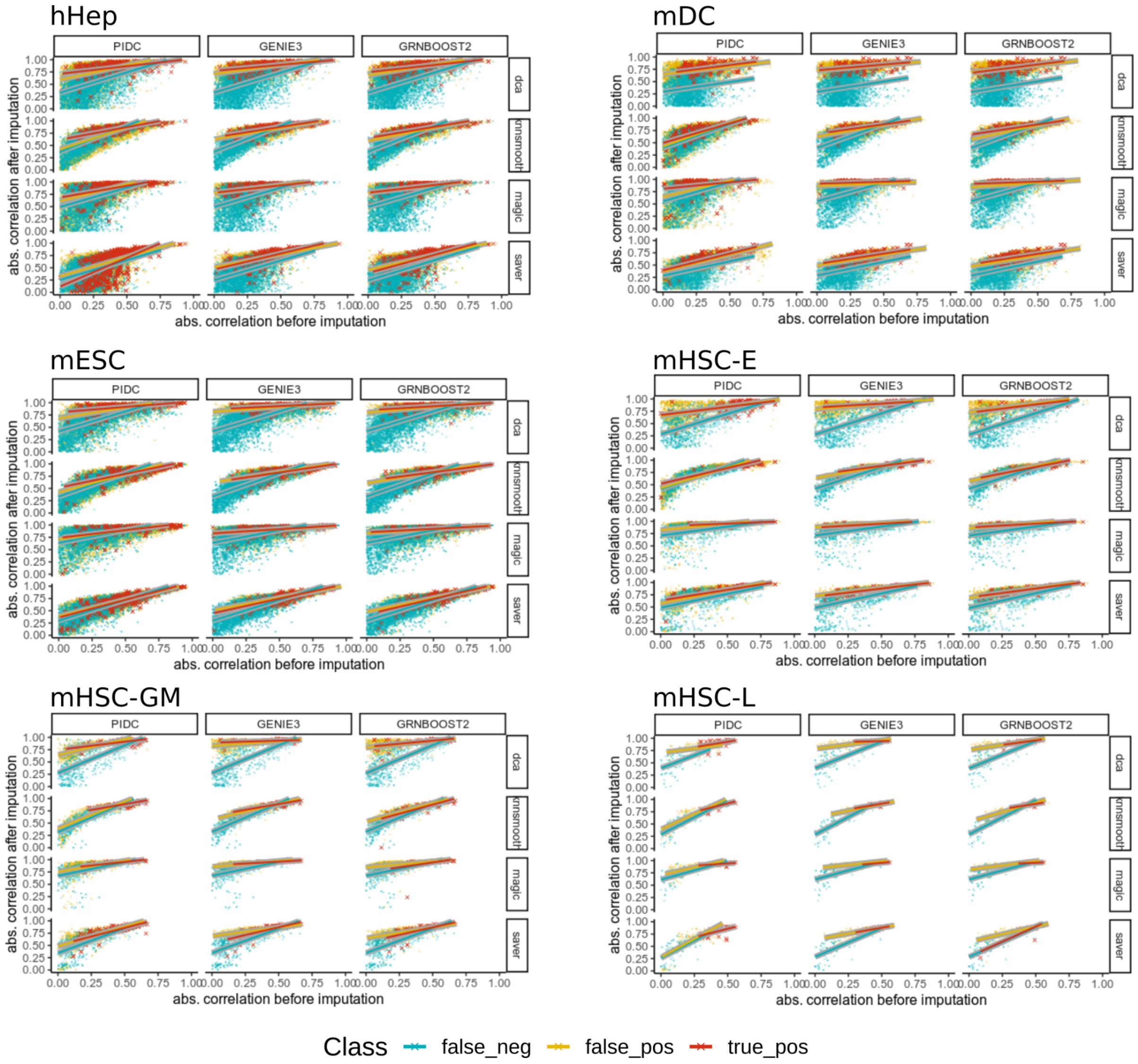

Fig. S7: Absolute Pearson's correlation coefficients before and after imputation colored by prediction class obtained in each model. Related to Figure 4D we inspect the change of correlation values for TP, FP and FN classified by each model in the remaining cell types. Colors correspond to the prediction classes in each model.

data	factor	sum of squares	mean of squares	p-value
hESC	GRN	0.0455	0.0228	0.68436
	imputation	2.3036	0.7679	0.00435 **
hHep	GRN	0.0205	0.0103	0.75904
	imputation	1.4728	0.4909	0.00421 **
mDC	GRN	0.0061	0.00307	0.68601
	imputation	0.3728	0.12428	0.00275 **
mESC	GRN	0.1324	0.0662	0.00638 **
	imputation	1.1478	0.3826	3.64e-05 ***
mHSC-E	GRN	0.1456	0.07281	0.07206 .
	imputation	0.6497	0.21657	0.00541 **
mHSC-GM	GRN	0.1748	0.08739	0.000247 ***
	imputation	0.5445	0.18151	2.02e-05 ***
mHSC-L	GRN	0.11682	0.05841	0.000572 ***
	imputation	0.08218	0.02739	0.003099 **

Tab. S1 | Analysis of variance of performance scores for each dataset. ANOVA results on EPR log-fold-ratios. Significance codes are 0 ‘***’ 0.001 ‘**’ 0.01 ‘*’ 0.05 ‘.’ 0.1 ‘ ‘ 0. Higher significance p-values in imputation give evidence that a higher variance within imputation methods compared to GRN algorithms is prevalent, and vice versa.

data	imputation	PIDC	GENIE3	GRNBOOST2
hESC	dca	6.23E-59	1.35E-52	3.22E-57
	knnsmooth	9.30E-70	5.56E-30	1.72E-35
	magic	5.41E-45	1.89E-47	1.68E-51
	saver	1.99E-15	5.46E-12	8.93E-13
hHep	dca	2.77E-144	3.67E-89	5.47E-83
	knnsmooth	1.11E-130	3.88E-85	5.87E-86
	magic	1.26E-126	1.19E-125	1.86E-108
	saver	1.66E-23	1.08E-37	1.34E-29
mDC	dca	1.15E-48	1.91E-39	8.93E-42
	knnsmooth	1.47E-46	5.11E-38	3.20E-38
	magic	2.14E-58	6.35E-31	9.12E-32
	saver	2.69E-10	7.78E-08	3.66E-10
mESC	dca	2.85E-75	6.53E-79	7.09E-83
	knnsmooth	2.84E-75	9.74E-59	5.33E-77
	magic	1.54E-78	1.25E-80	4.75E-90
	saver	3.61E-05	3.28E-07	7.37E-10
mHSC-E	dca	1.93E-08	2.97E-24	6.55E-25
	knnsmooth	1	4.70E-07	1.72E-10
	magic	2.44E-14	6.80E-27	4.39E-26
	saver	1	0.001687	0.0004226
mHSC-GM	dca	5.32E-44	1.02E-33	7.75E-32
	knnsmooth	0.05846	2.32E-09	9.27E-17
	magic	8.66E-21	2.31E-23	2.89E-22
	saver	1	8.45E-07	6.18E-09
mHSC-L	dca	1	0.006637	1.51E-05
	knnsmooth	1	1	0.3427

	magic	0.005388	0.004132	3.54E-06
	saver	0.7522	1	0.0006845

Tab. S2: Statistical testing of differences between edge rank distributions. Corrected p-values (Bonferroni method) obtained after Wilcoxon rank sum test between ranks of unimputed true positive edges and their respective ranks after imputation (see Fig. 4C, Fig. S6).
