# Supplementary material for: Effect of imputation on gene network reconstruction from single-cell RNA-seq data
Source: Patterns (N Y). 2021 Dec 22;3(2):100414. doi: 10.1016/j.patter.2021.100414 (PMC8848013; doi:10.1016/j.patter.2021.100414)
Supplement: Document S2. Article plus supplemental information [file mmc3.pdf]

Article

Patterns

Effect of imputation on gene network reconstruction from single-cell RNA-seq data

Highlights

- Gene network reconstruction does not necessarily profit from imputation
- Imputation rather than network reconstruction method influences network result
- Inflation of enhanced gene-gene correlations can obscure inferred network structures

Authors

Lam-Ha Ly, Martin Vingron

Correspondence

vingron@molgen.mpg.de

In brief

Data analysis for single-cell transcriptomics requires sophisticated software pipelines. However, individual components of such a pipeline may be interdependent, and this interdependence is frequently overlooked. In this study, we investigate the interplay between an early step in single-cell transcriptome analysis, the imputation of missing data, and the later task of gene network reconstruction. We demonstrate that the choice of imputation method strongly influences the possible predicted network structures.

Ly & Vingron, 2022, Patterns *3*, 100414
February 11, 2022 © 2021 The Author(s).
https://doi.org/10.1016/j.patter.2021.100414

CellPress

Patterns

CellPress
OPEN ACCESS

Article

Effect of imputation on gene network reconstruction from single-cell RNA-seq data

Lam-Ha Ly[1] and Martin Vingron[1,2,*]
[1]Department of Computational Molecular Biology, Max Planck Institute for Molecular Genetics, 14195 Berlin, Germany
[2]Lead contact
*Correspondence: vingron@molgen.mpg.de
https://doi.org/10.1016/j.patter.2021.100414

THE BIGGER PICTURE Data analysis for single-cell transcriptomics requires sophisticated software pipelines. By studying the interplay between two prominent tasks, imputation of missing data, and gene network reconstruction, we point out the pitfalls of freely combining components as part of an analysis pipeline. In our application, an earlier decision for a particular imputation algorithm is shown to largely determine the results achievable in the later gene network reconstruction task. This interdependence constitutes the flip side of the convenience that comes with the availability of user-friendly computational pipelines.

1 2 3 4 5 **Proof-of-Concept:** Data science output has been formulated, implemented, and tested for one domain/problem

SUMMARY

Despite the advances in single-cell transcriptomics, the reconstruction of gene regulatory networks remains challenging. Both the large amount of zero counts in experimental data and the lack of a consensus preprocessing pipeline for single-cell RNA sequencing (scRNA-seq) data make it hard to infer networks. Imputation can be applied in order to enhance gene-gene correlations and facilitate downstream analysis. However, it is unclear what consequences imputation methods have on the reconstruction of gene regulatory networks. To study this, we evaluate the differences on the performance and structure of reconstructed networks before and after imputation in single-cell data. We observe an inflation of gene-gene correlations that affects the predicted network structures and may decrease the performance of network reconstruction in general. However, within the modest limits of achievable results, we also make a recommendation as to an advisable combination of algorithms while warning against the indiscriminate use of imputation before network reconstruction in general.

INTRODUCTION

Single-cell transcriptomics has revolutionized genomics. In particular, this new type of data is widely assumed to advance the unraveling of regulatory interactions in the cell. Thus, there is great interest in the computational reconstruction of gene regulatory networks (GRNs) from single-cell transcriptome data.

Available methods for GRN reconstruction from single-cell RNA-seq (scRNA-seq) data draw on a plethora of statistical approaches.[1–6] Pratapa et al.[4] provide an extensive benchmark study evaluating the performance of various methods. However, for GRN reconstruction, several authors have remarked that preprocessing the data is important, mostly due to the sparse nature of the data.[7,8] Several computational analysis pipelines have been suggested and are in wide use.[9,10] Typically, as one of the early steps, such a pipeline will include a data normalization and/or imputation step, which statistically estimates unobserved read counts in cases where the method deems that experimental or technical noise has led to the absence of a count (i.e., a so-called dropout). While normalization attempts to correct for different read depths between cells,[11,12] imputation attempts to recover gene counts by predicting missing data and eventually smoothen gene expression values.[13–20] In some tools, a prior normalization step is not required but is integrated within the imputation method.[15,18] Hou et al.[21] extensively evaluated the impact of imputation on clustering, differential expression analysis, and pseudotime inference and invoked cautious interpretations of the results.

It still remains unclear, though, how imputation methods affect network structures.[22] On the one hand, it is recommended to use imputation to enhance gene regulatory correlations prior to network inference[14,15], but on the other hand, results based on

This is an open access article under the CC BY license (http://creativecommons.org/licenses/by/4.0/).

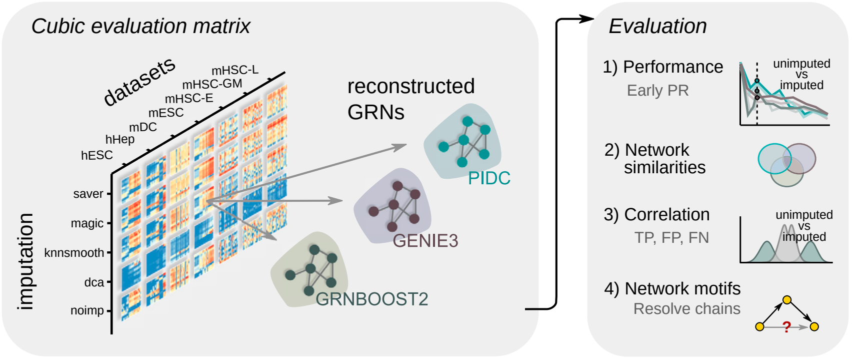

Figure 1. Systematic evaluation of network reconstruction from imputed and unimputed data

Cubic evaluation matrix consists of seven cell types from experimental scRNA-seq data, four imputation methods (see text), and three network reconstruction algorithms. Imputed and unimputed (noimp) scRNA-seq data provide input expression matrices, which are used by the GRN reconstruction algorithms using the BEELINE framework.[4] We evaluate the performances using the EPRs and compare network results across different models. Additionally, we inspect the effect of gene-gene correlation on prediction classes (TPs, FPs, FNs) before and after imputation, and we search for common motifs within the reconstructed networks.

imputed data should be interpreted with care.[9,21,23] Thus, imputation meets conflicting attitudes within the community.

Here, we systematically study the question of whether data imputation as a preprocessing step affects results obtained using reconstructed GRNs. We build on previously published benchmark studies and consider the best-performing scRNA-seq-based tools for both imputation and network reconstruction in our analysis. We measure the performance of different combinations of imputation method and GRN reconstruction method using multiple experimental datasets and evaluation networks that have been used in other benchmark studies. We compare the performance and network structures obtained using unimputed data and imputed data, respectively, and show that, in most cases, GRN reconstruction does not profit from imputation. In order to explain the observed results, we analyze the effect of imputation on predicted gene interactions. Ultimately, we present a recommendation on how to proceed in a data analysis project.

RESULTS

Systematic evaluation of network models

Evaluating the combination between imputation and network inference on different datasets results in a cubic matrix. To manage this, we restrict our selection to state-of-the-art computational tools, both for imputation and network inference, that perform most accurately and have been recommended in recent benchmark studies.[4,21] Consequently, we developed a computational pipeline to study seven cell types that were obtained from different scRNA-seq experiments, using four state-of-the-art imputation methods combined with the top-performing GRN methods, as depicted in Figure 1.

Information on the seven cell types was derived from five experimental scRNA-seq datasets: human embryonic stem cell (hESC),[24] human hepatocyte (hHep),[25] mouse embryonic stem cell (mESC),[26] mouse dendritic cell (mDC),[27] and mouse hematopoietic stem cell (mHSC).[28] These were further separated into the following subtypes: erythrocyte (mHSC-E), granulomonocyte (mHSC-GM), and lymphocyte (mHSC-L). We preselected the datasets according to significantly varying transcription factors (TFs) and the most highly variable genes across pseudotime (see experimental procedures).

For the four imputation methods, we chose the following methods, summarized in Table 1: two smoothing-based tools, *magic*[14] and *knnsmooth*[20]; a Bayesian model-based tool *saver*[16]; and a deep-autoencoder-based tool *dca*.[15] We included *dca* because the authors specifically expect to improve network reconstruction. A baseline model was established using normalized but unimputed data.

As for GRN reconstruction, we selected the following tools: an information-based tool PIDC,[2] and two tree-based tools, GENIE3[29] and GRNBoost2.[30] The PPCOR[31] method is based on partial correlations and, as such, also a contender for a good network reconstruction method. However, PPCOR results on single-cell data are clearly inferior to those obtained with any of the first three methods, as shown in Figure S1. While we have included PPCOR in this performance comparison, we focus the study of the relationship between imputation and network reconstruction on the other three methods. Table 2 gives an overview about the underlying concepts and assumptions of each GRN algorithm.

In the remainder of this paper, we use the term "model" to refer to the combination of a GRN reconstruction algorithm with an imputation method or no imputation, respectively. We obtain the evaluation networks from the STRING (Search Tool for the Retrieval of Interacting Genes/Proteins) database, a functional protein-protein interaction network,[32] as well as cell-type-specific chromatin immunoprecipitation sequencing (ChIP-seq)-derived networks provided by Pratapa et al. Studying gene regulation, we only consider edges outgoing from TFs in the reconstructed networks. To evaluate the performance of each network model, we use the evaluation framework BEELINE[4] (see experimental procedures). Furthermore, we inspect the reconstructed network and compare the results with one another.

Imputation does not improve the performance of network reconstruction in general

A compact overview of the results obtained under all the models compared with the STRING network is provided in Figure 2. Analyses have been performed on sets of significantly varying TFs along with 500 and, respectively, 1,000 most highly variable genes (HVGs; see experimental procedures). Each box summarizes results for one GRN reconstruction method. The performance measurements achieved by the respective model on the seven cell types are arranged on a vertical axis. Two performance measures have been computed for each prefiltered gene set correspondingly: the early precision ratios (EPRs),[4] which are shown in the three boxes of Figure 2A, and the log2 ratios

Table 1. Overview of selected imputation methods

Imputation methods	Concept	Distributional assumption	Gene-gene relation predictions
dca	deep autoencoder	negative binomial distribution with or without zero inflation	non-linear
knnsmooth	kNN graph, smoothing by aggregation	negative binomial distribution	linear
magic	kNN graph, smoothing by diffusion	low rank representation (no data assumption)	non-linear
saver	bayesian modelling	negative binomial distribution	linear

Imputation methods are summarized based on their underlying concept. The distributional assumption is used to reduce the noise in the count data. The predictions with regard to gene-gene relation can be either non-linear or linear.

between $EPR_{imputed}$ and $EPR_{unimputed}$, which are shown in the three boxes of Figure 2B. EPR refers to the number of true-positive (TP) interactions within the top-k network normalized by the network density. Here, *k* refers to the number of positive interactions found in the evaluation network (see experimental procedures). An EPR of 1 indicates a random predictor. The second performance measure compares the performance of an imputation method relative to the performance of not using imputation. Here, a value of zero means no change, while a negative value indicates a detrimental effect of the imputation.

The EPR scores for unimputed data that were reported by Pratapa et al.[4] could be reproduced with minor deviations in our analysis. The EPR scores are illustrated as a dashed line in Figure 2A (Data S1). Results vary strongly with the datasets; the scores range from approximately 2 (for the mDC dataset) to 8 (for mHSC-GM), with less variation across GRN reconstruction algorithms. Applying imputation with either *dca*, *knnsmooth*, or *magic* does not improve the performance in any of the GRN reconstruction methods. While, in mDC data, the performance scores in each model scatter around the unimputed model, in mHSC-GM data the performance scores vary strongly, dropping from 8 to just below 5 for the *magic*/GENIE3 model. As pointed out above, for PPCOR we observe considerably lower performance scores compared with the remaining GRN algorithms (Figure S1). The respective EPR scores indicate predictions so close to a random model that we decided to exclude PPCOR from further evaluations.

Focusing on the change of performance due to imputation as measured using the log2 ratios between imputed and unimputed EPR scores, we observe that only *saver* is able to improve the performance (Figure 2B). The *saver*/PIDC model achieves log-fold ratios up to +0.5 in five out of seven datasets and two out of seven datasets combined with GENIE3 or GRNBoost2. All other imputation methods worsen the performance with log-fold ratios down to -1, which represents a performance decline of 50% in comparison with the unimputed model. Generally, the performance results regarding the number of most HVGs are highly consistent, suggesting that the predictions are irrespective of the number of genes selected as an input.

Furthermore, we use cell-type-specific networks derived from ChIP-seq data as an evaluation network (Figure S1). Here, absolute EPR scores report very poor performances close to or worse than a random predictor regardless of the model or the number of input genes across all datasets. Thus, the ChIP-seq network does not serve us well for distinguishing between methods in terms of their accuracy. The STRING database, on the other hand, may contain indirect interactions reported in the protein-protein interaction data. We will return to this issue below in the context of network motif analysis. Nevertheless, independent of the evaluation network, we do not see an improvement of GRN reconstruction if imputation has been used in advance.

We further asked whether data quality as given by sequencing depth is a determinant of the success of imputation prior to GRN reconstruction. To answer this, we simulated cells *in silico* by downsampling the gene counts of the given experiments to 60% of their sequencing depth, thereby lowering the detection rate (Figure S2). The hope would be that imputation has a more beneficial effect in these simulated datasets compared with the original, higher-quality data. However, similar results to those above were obtained when we subjected the lower-quality *in silico* data to our analysis pipeline (Figure S3). As with the original datasets, *saver*/PIDC obtain the highest improvements compared with the downsampled unimputed datasets. Nonetheless, on downsampled data, *dca*, *knnsmooth*, and *magic* are able to improve performance in some of the tested datasets, although not consistently.

Overall, our results demonstrate that model performances are highly dataset dependent. Applying imputation on the original data resulted mostly in a drop of performance of GRN reconstruction compared with the unimputed model, although potentially improving performance on low-quality data tested *in silico*.

Imputation method rather than GRN method determines results

The analysis presented in the preceding section raises the question of how strongly either the choice of imputation method or of network reconstruction algorithm affects the results. To answer this question, we first address the variability in results when varying either the one or the other, and then study similarity among computed networks across the models.

With regard to the performance variability, we compare the variance of EPR log-fold ratios under a fixed GRN reconstruction algorithm while varying across imputation methods, and, vice versa, varying the GRN algorithm while keeping the imputation method fixed. As Figure 3A shows, EPR log-fold ratios vary much more strongly across the imputation methods than across GRN methods (Wilcoxon test p value $\sim 7.86 \times 10^{-6}$). Since this analysis aggregates all datasets jointly, it discards the differences between datasets. Comparing, e.g., hESC and mHSC-L, we see large differences between the distributions of variances across imputation methods and GRN algorithms, respectively. To resolve this, we perform an analysis of variance (ANOVA) with respect to the EPR log-fold ratios for each dataset separately. The results give evidence that imputation has a larger contribution to the variance of performance scores compared

Table 2. Overview of selected network reconstruction algorithms

GRN algorithms	Concept	Assumptions about gene-gene relation	Data prerequisite	Edge predictions
PIDC	mutual information	possibly non-linear	discrete	undirected
GENIE3	tree-based	possibly non-linear	continuous	directed
GRNBoost2	tree-based with gradient boosting	possibly non-linear	continuous	directed
PPCOR	partial correlation	Gaussian, linear	continuous	undirected

GRN algorithms are summarized based on their underlying methodology and theoretical assumptions about gene-gene relation. Except PPCOR, the algorithms do not have strong assumptions about gene-gene relation, and thus non-linear interactions can possibly be inferred. PIDC requires a data discretization, whereas all other methods work on continuous data. The inferred network may contain causal interactions (directed) or associated interactions (undirected).

with GRN algorithms, prevalent in all datasets except mHSC-L (Table S1). This implies that the choice of imputation method determines the quality of results to a larger degree than the choice of GRN reconstruction algorithm.

A direct consequence of this observation is the suspicion that the topology of the predicted networks may also be largely determined by the imputation method and, to a lesser degree, by the GRN reconstruction method. To test this, we inspect the overlap among the 500 most important gene-gene interactions of the computed networks. Here, we calculate pairwise similarity scores using the Jaccard index and use them to hierarchically cluster the networks. We found that networks tend to cluster with respect to imputation methods but not GRN methods (Figures 3B and S4). To make this more precise, we use as a measure of cluster purity the adjusted Rand index (ARI).[33,34] ARI coefficients calculated across the seven different cell types show higher cluster purity when labelled with imputation methods as opposed to network reconstruction algorithms (Figure 3C).

We conclude that the imputation method largely determines model performance, leaving little influence to the subsequent GRN reconstruction algorithm. The choice of imputation method further biases the outcoming network, leading to little consensus across the most important recovered gene-gene interactions as computed based on different imputation methods.

Inflation of gene-gene correlations and its impact on the network topology

Based on the reported results, we examine how imputation generally affects gene-gene correlation coefficients. Although not all network reconstruction algorithms use correlation-based measures to recover interactions, we still use Pearson's correlation coefficient as a proxy for the association between two genes. Subsequently, we will investigate whether the interactions within the reconstructed networks affect the global network structure.

Exploring the overall distributions of gene-gene correlations after imputation on scRNA-seq data we observe a strong enhancement in gene-gene correlations (Figure 4A). Generally, gene-gene correlations go from almost no correlation when computed using unimputed data to very good anti- and positive correlations due to imputation. Here, *magic* leads to the most extreme enhancement. Surprisingly, even the unimputed distribution within the mDC data is skewed toward positively correlated values. We have checked that this is not due to selection of the most HVGs but rather is already present in the dataset. Figure 4B exemplifies the association between three genes before and after imputation, transforming very weak correlations to almost perfect (anti-)correlations. This particular set of gene interactions was observed in the top-k network computed with GRNBoost2 on hESC data, comparing no imputation with *dca* imputation. Indeed, we commonly find such associations across different datasets and imputation methods.

In order to see what impact this enhancement of correlation has on the network structure, we next investigated the network density after imputation in relation to the unimputed data using log ratios (Figure S5A). Here, we looked at the top-k networks according to the EPR score. Imputation methods alter the network densities with log ratios ranging from -0.5 and $+0.5$ in hESC, hHep, mDC, and mESC data, except for *saver* and PIDC in hESC data with a slightly higher value of 0.59. For the three subtypes of mHSC data, we observe larger changes in network density reaching log ratios beyond ± 1. Especially here, imputations combined with GENIE3 and GRNBoost2 lead to a sparser network, whereas all combinations of imputation methods with PIDC lead to a denser network structure. We assume that this is due to redistribution of edges occurring in the tree-based algorithms, which is also reflected in the node degree distribution (Figure S5B).

Before imputation, we observe a heavy tail node degree distribution predominantly in GENIE3 and GRNBoost2 indicating the presence of many hub nodes. After imputation, the heavy tail disappears when using *dca*, *magic*, and *knnsmooth*, while it still exists when using *saver*. Generally, PIDC does not lead to this structural change in node degree distribution.

As a conclusion, the enhancement of gene-gene correlations due to imputation appears to lead to notable changes in the topology of the predicted gene networks.

Increased correlation values lead to inflation of false-positive predicted interactions

Since we have observed that imputation may decrease the performance of GRN network reconstruction, we attempt to understand how the altered correlations in imputed data affect network reconstruction. To this end, we explore the change of edge ranks and correlation values of the reported (i.e., positively predicted) and missed (i.e., negatively predicted) interactions.

Overall, the ranks of TP interactions reported in the unimputed data change significantly after imputation (Figures 4C, S6, and Table S2). Some of the previously reported TP interactions could be recovered after imputation. Nevertheless, the majority of previously reported TP interactions shift after imputation toward the end of the gene interaction ranking list, and are considered less

CellPress
OPEN ACCESS

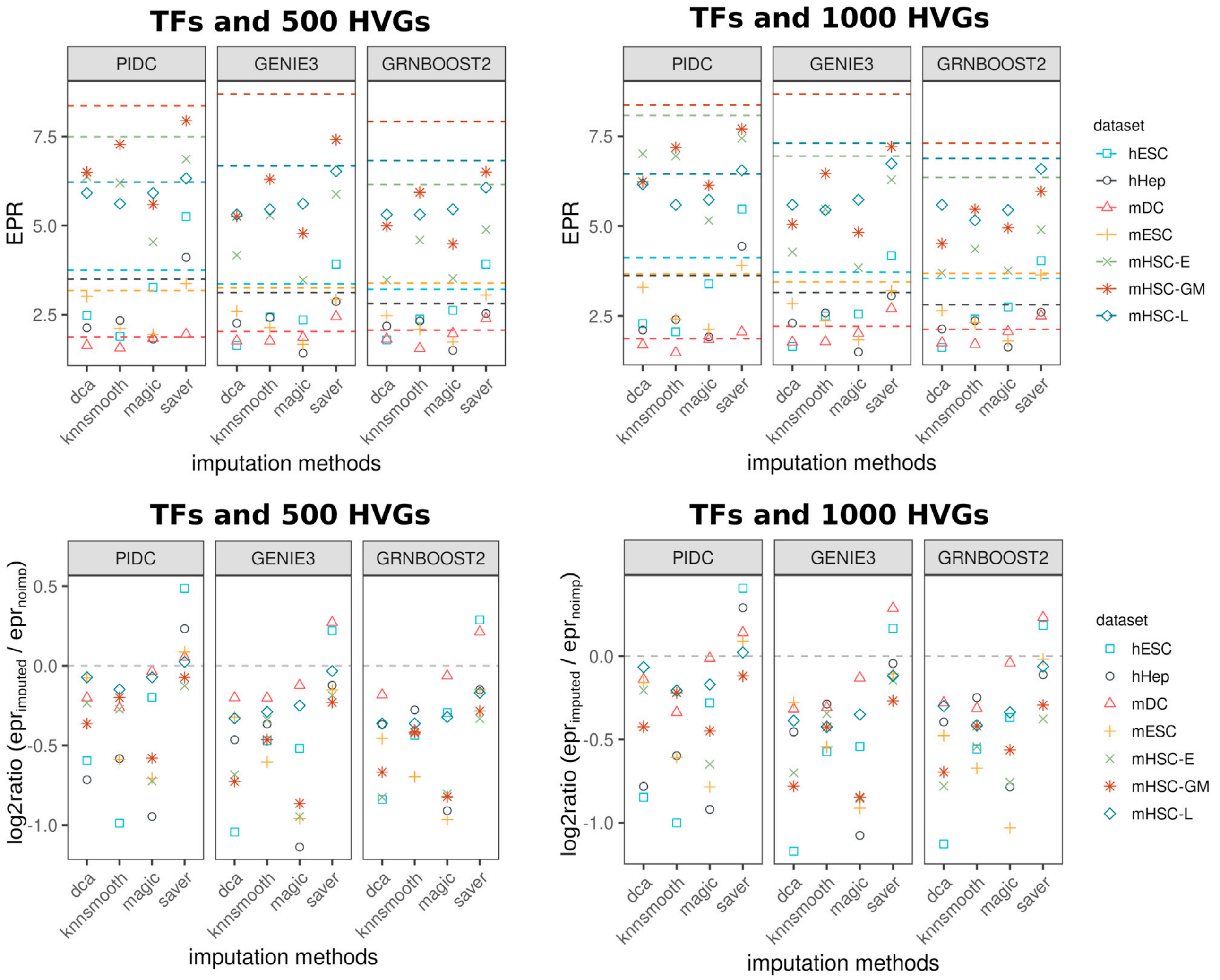

Figure 2. Impact of imputation on network reconstruction performances

Results under all models with two different sets of genes compared with the STRING network.

(A) Absolute EPR scores across imputation methods (x axis label) and GRN inference algorithms (box) on seven different cell types (coded by shape and color). Dashed lines represent EPR scores obtained without imputation. EPR = 1 corresponds to a random predictor.

(B) log2 ratios between EPR scores obtained using imputed and unimputed data. Log2 ratio = 0 represents no change in performance (gray dashed line) after imputation.

important. As a consequence, other interactions become more important.

Therefore, we look at the change of correlation of positively predicted interactions before and after imputation. Figure 4D (and Figure S7) show scatterplots of gene-gene interactions with the absolute values of correlation coefficients before imputation on the horizontal axis and the correlation coefficient after imputation on the vertical axis. For each model, red dots are the TP interactions, yellow are the false-positives (FPs), and blue are the false-negatives (FNs). The general shape of the scatterplot reiterates the observation that correlation coefficients tend to get enhanced by imputation. For each class we computed regression lines. For better recognition of TPs after imputation, one would hope for the TP regression line (shown in red in Figure 4D) to lie well above the others, which is not really the case. We generally observe a strong enhancement of correlations as indicated by the height of the intercept of the regression lines. In 11 out of 12 cases, the regression lines for both TP and FP predictions are almost congruent with each other. Note that the red color dominates the other ones and the dots below a red one are not visible.

Interestingly, we see remarkably different regression lines if we take the FN (blue) interactions into account. The majority of FN correlations remain low after imputation, as indicated by the height of the intercept in Figure 4D. Presumably, the FN correlation values that actually get enhanced get lost in the background due to the inflation of FP correlations in the inferred top-k network. Thus, the boost of correlation values makes it harder for GRN reconstruction methods to separate the actual signal from the background.

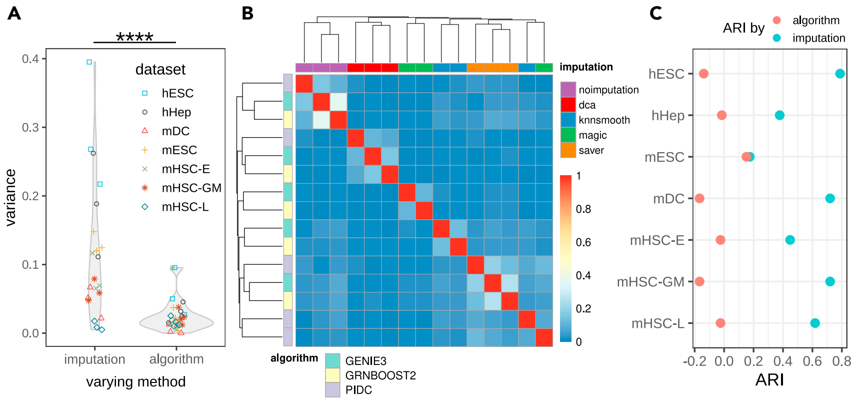

Figure 3. Variability in network results largely stems from imputation methods

(A) Variance distribution of EPR scores across imputation methods. Left violin plot keeps the GRN algorithm fixed and depicts the variances in EPR log-fold ratios for each dataset across the imputation methods. Right violin plot shows the variances for fixed imputation methods. ****$p \leq 0.0001$ by Wilcoxon rank sum test.

(B) Clustered heatmap of network similarities measured by Jaccard index within top 500 reported interactions. Columns (horizontal axis, above) are color coded by imputation methods. Rows (vertical axis, left) are color coded by network inference algorithms. More pure clusters are obtained by imputation than by GRN algorithm.

(C) ARI obtained for clustering results in each cell type by annotation label algorithm (pink) and imputation (blue), respectively.

Many GRN reconstruction methods have the goal of distinguishing direct interactions from transitively inferred ones.[35] Therefore, we tested whether the GRN reconstruction algorithms analyzed in this study are able to make the necessary distinction. Given three genes X, Y, and Z, where X is correlated with Y, and Y is correlated with Z, these genes form a network chain. However, oftentimes by transitivity these associations seem to imply a correlation between X and Z, thus forming a network loop. Generally, in network theory, it is challenging to distinguish chains from loops, and algorithms deal differently with it. PIDC constrains the inferred interactions based on a confidence score to discriminate between direct and indirect interactions. GENIE3 and GRNBoost2 allow the user to set a parameter for filtering out presumably indirect interactions. In this context, we analyze how the models deal with the identification of network chains from imputed data among the top-k networks. Errors are counted if a supposedly false loop is detected (FN) or a chain is detected instead of a loop (FP). However, STRING is less suited as an evaluation network in this context because FP counts might be overestimated upon comparison with STRING. This is due to the fact that the STRING database is not designed to contain only direct interactions. For example, protein complexes are reported in STRING and may contain indirect associations. Therefore, in this analysis we use the ChIP-seq-derived networks as the more appropriate evaluation networks. Figure 4E shows TP counts together with the error counts in hESC data. Here, we observe mainly lower motif counts after imputation. In general, low count numbers in the motif search are indicative of isolated edges between a gene pair. Hence, the algorithms detect fewer connected edges among the top-k networks.

In order to measure the performance between true and false predictions, we also calculate the TP rates (TPRs) and false discovery rates (FDRs) for each network inference and imputation method applied to each dataset (Figure 4F). Ideally, the values for TPR should be higher (yellow), while the values for FDR stay low (purple). Comparing the TPR and FDR scores after imputation, however, we do not see systematic differences. We conclude from this observation that the performance of network motif detection among the top-k networks does not seem to be affected by imputation. Hence, either imputation methods do not necessarily induce transitive correlations or the network reconstruction methods deal well with transitively induced correlations.

DISCUSSION

The advent of single-cell transcriptomics has rekindled the interest in reconstructing GRNs from transcriptomics data, primarily for two reasons. First, it is of great interest to study regulation from single-cell data in the hope to eventually uncover how, e.g., differentiation processes proceed. Second, the main obstacle in gene network reconstruction from bulk transcriptome data appears to be the low number of available samples in comparison with the large numbers of genes. For example, simulations have demonstrated that high-quality reconstruction of gene networks requires a much larger number of samples than the number of genes.[35] Seeing each single cell as a sample, the expectation arose that single-cell transcriptomics would solve this conundrum by providing a sufficiently large number of samples, thus putting high-quality network reconstruction within reach.

It was sobering for us to see that, due to the sparse nature of scRNA-seq data, individual cells cannot contribute as much information to network reconstruction as bulk samples. Indeed, preprocessing of single-cell data for data analysis is crucial,[10] and is implemented in many computational pipelines. Imputation has become a possible element of this preprocessing in the hope it would supplement the missing information. In this study we have, however, demonstrated that the choice of imputation prior to GRN reconstruction influences the results in a 2-fold manner: first, it affects the performance of network reconstruction, leading to highly variable accuracies, and, second, the reconstructed network is determined more by the imputation method than by the choice of network reconstruction method.

The focus of our work on the interplay of the imputation step with GRN reconstruction clearly also limits the scope of our work: we have not attempted to compare GRN methods as such, or to improve GRN reconstruction. Many other publications are dedicated to these issues, with GRN reconstruction being a particularly hard problem, as shown by the overall meager results that can be obtained.[4,36] Still, what has clearly been understudied is the interdependence between imputation, which is routinely done in single-cell data analysis, and GRN reconstruction.

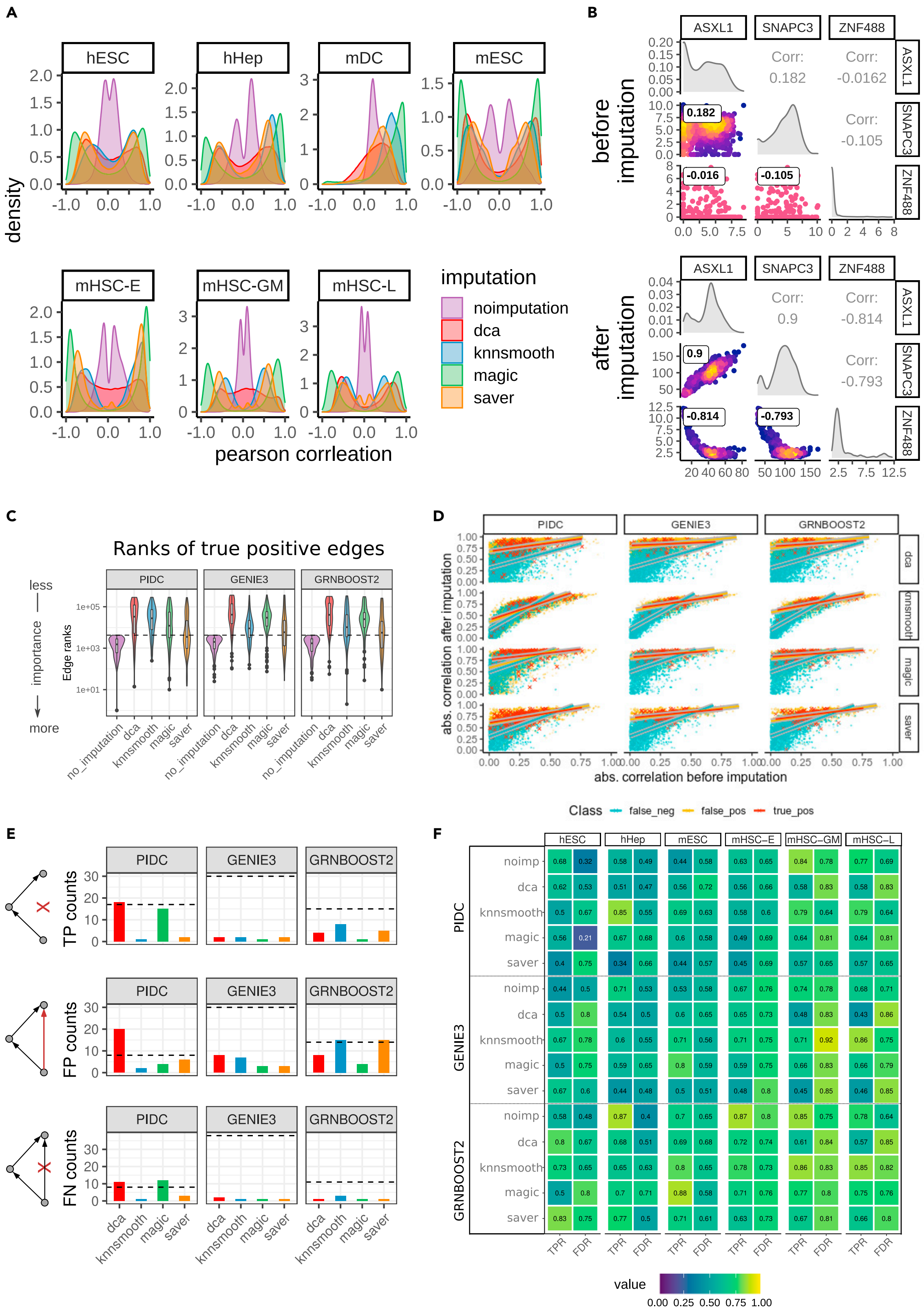

Figure 4. Gene-gene correlation before and after imputation and its impact on the predicted interactions

(A) Gene-gene correlation distributions obtained in each cell type color coded by imputation method among top 500 most variable genes and significantly varying TFs.

(legend continued on next page)

We have systematically evaluated the effect of imputation on GRN reconstruction using experimental scRNA-seq data on seven cell types. In agreement with previous studies, we see that imputation may boost gene-gene correlations in a questionable way, thereby introducing FP edges in a network.[23,37] Steinheuer et al.[37] evaluated the impact of data imputation on network inference via a gene correlation analysis using simulated data. There, the authors downsampled bulk RNA-seq data, applied imputation methods, and compared the gene module preservation and edge recovery upon imputation. Similar to our observation, they noticed a higher number of FP interactions after imputation.

We have provided evidence that these FP may lead network algorithms to reinforce dependencies that have been introduced by imputation. For example, regression-based methods like GENIE3 and GRNBoost2 will be strongly predisposed to including imputation-generated correlations into a network. Table 1 recalls which assumptions imputation methods make with respect to signal distribution and the linear or non-linear nature of interactions. Likewise, GRN reconstruction algorithms are each based on their own respective assumptions (Table 2). This may lead to reinforcement of imputation decisions or, generally, to the identification of wrong gene-gene dependencies. Andrews et al. have warned of this circularity before, albeit in the context of differential expression analysis.[38] Consistent with our findings, Andrews et al. showed that *saver* introduces the smallest number of spurious gene-gene correlations. We speculate that the combination of *saver*/PIDC works well because *saver* is a model-based imputation method and PIDC is a mutual-information-based algorithm discretizing the data beforehand; the two approaches follow independent assumptions complementing one another, thus avoiding the use of redundant information.

In this study, we have tested our hypothesis on experimental datasets with fairly large library sizes and gene detection rates (Figure S2). In order to test our hypothesis on more shallowly sequenced single-cell experiments, we *in silico* lowered the detection rate, introducing more zero counts. These results again show that using *saver* with PIDC improves results in most cases. Thus, if single-cell data are too sparse to avoid imputation altogether, we recommend the use of *saver* and PIDC. It should be noted, though, that we are not discouraging imputation in general. There may be many other applications that are not studied here, where imputation can be useful, depending on the type of analysis that is subsequently performed.

We believe that the described interdependence among processing steps within a data analysis pipeline is exemplary for many data analysis tasks. Software is generally being built to allow the user to freely combine algorithms, each dedicated to a particular step of the analysis. Little attention is given to the possible influences one algorithm might have on the behavior of the other. We are not referring to a syntactic interaction in terms of data structures or variables passed, since good, modular software design will exclude such conflicts between processing steps. Much rather, as we demonstrated for imputation and GRN reconstruction, decisions taken within one algorithm may predispose the results that can be obtained in a downstream analysis step. Thus, user friendliness in pipeline design allowing the free combination of algorithms may carry substantial risk with respect to the scientific validity of data analysis results.

Limitations of the study and future insights

The findings of the study presented have some limitations that we want to address here. Above all, the wiring of the cell is still not fully understood and thus the choice of gold-standard dataset for GRN reconstruction will necessarily remain problematic. While considering TF-based gene regulation, we follow the literature in that we use STRING and cell-type-specific datasets of ChIP-seq-derived interactions for evaluation. However, all methods studied have difficulties in identifying interactions from the latter dataset. Thus, we learn more from the STRING database, although it contains indirect interactions and does not contain cell-type-specific information.

There is further room for improvement in exploiting pseudotime derived from single-cell data. However, the methods geared toward this goal follow different principles, and Pratapa et al.[4] have shown that network reconstruction algorithms using pseudotime information are very sensitive to the temporal ordering of the cells. Thus, in addition to studying the dependence between imputation and GRN reconstruction, it would also be necessary to study the interplay between pseudotime reconstruction method and GRN reconstruction. The preeminent question following from our study is clearly how one can best utilize the large number of cellular transcriptomes for the purpose of GRN reconstruction without initially relying on imputation.

EXPERIMENTAL PROCEDURES

Resource availability

Lead contact

Further information and requests for resources should be directed to and will be fulfilled by the lead contact, Martin Vingron (vingron@molgen.mpg.de).

(B) Paired density scatterplots before and after imputation with *dca*. GRNBoost2 reported the pairwise interactions between ASXL1, SNAPC3, and ZNF488 among the top-k network after imputation in hESC data.

(C) Change of edge ranks in TP interactions identified by unimputed model after imputation in hESC data. Dashed line indicates the rank threshold corresponding to the top-k network. Interactions below the dashed line represent TP within the respective model. Low edge ranks represent highly important interactions.

(D) Scatterplots comparing correlation values between genes before versus after imputation. Each scatterplot corresponds to one model in hESC data. TPs are red crosses, FPs are yellow dots, and FNs are blue dots. For each scatterplot, we fit one regression line for TPs, FPs, and FNs, respectively, with the corresponding color. For visualization purposes, we added a background color to the lines to better distinguish the line and the dots. Positively predicted interactions differ clearly from FN interactions.

(E) Counts of positively and negatively predicted network chain motifs in hESC data for each model. TP network chains agree both in prediction and evaluation networks (ChIP-seq-derived network). FP network chains are falsely positively predicted chains being actual feed-forward loops in the evaluation network. FN network chains are falsely predicted as being feed-forward loops when they are actually network chains in the evaluation network.

(F) TPR and FDR scores for network chain motifs obtained by statistics in (E). mDC dataset is not included as no motifs could be found among the top-k network. Ideally, TPR values should be close to 1, whereas FDR values should be close to 0.

Materials availability

This study did not generate new unique reagents.

Data and code availability

This paper analyzes existing, publicly available data. Please see the section "data collection and preprocessing of scRNA-seq data" for more details.

All original code has been deposited at Zenodo under the DOI https://doi.org/10.5281/zenodo.5710368 and is publicly available as of the date of publication. The release includes tutorials from data imputation to the evaluation of the reconstructed networks. It covers the evaluation pipeline with the corresponding analyses and plotting results.

Any additional information required to reanalyze the data reported in this paper is available from the lead contact upon request.

Data collection and preprocessing of scRNA-seq data

We collected preprocessed and normalized experimental scRNA-seq count data provided in the BEELINE paper.[4] Here, the authors also provide the corresponding pseudotime for each dataset/cell type. Please refer to the BEELINE paper for information about preprocessing, normalization, and pseudotime inference.

However, *dca* needs unnormalized raw count data. Therefore, we downloaded the fastq files using the corresponding accession numbers: GSE75748 (hESC),[24] GSE81252 (hHEP),[25] GSE98664 (mESC),[26] GSE48968 (mDC),[27] and GSE81682 (mHSC).[28] For human and mouse, we aligned the fastq files to hg19 (GENCODE release 29) or mm10 (GENCODE release M19), respectively, and counted the reads per gene using STAR (version 2.7.4a).[39]

Following the BEELINE approach, using normalized count data, we select the top 500 most variable genes (or top 1,000 most variable genes, respectively) across pseudotime using a general additive model (gam R package). In addition to these genes, we also include significantly varying TFs (Bonferroni corrected $p < 0.01$).

We filter both imputed and unimputed scRNA-seq data using the same set of (1) top 500 most variable genes (or top 1,000 HVGs) and (2) all significantly varying TFs, in order to make a fair comparison between networks inferred using imputed and unimputed data.

Imputation

To impute scRNA-seq data, we use *dca* (version 0.2.3), *knnsmooth* (version 2.1), *magic* (Rmagic R package version 2.0.3), and *saver* (SAVER R package version 1.1.2). Our rationale for selecting *knnsmooth*, *magic*, and *saver* is based on a previous comprehensive benchmark evaluation of various imputation methods.[21] Additionally, we also include *dca* as it has been explicitly recommended as improving GRN reconstruction.[15]

We apply each imputation method to normalized count data, except for *dca*, where we use the raw counts (see github page).

Network reconstruction via BEELINE

Several tools have been developed to infer GRNs from scRNA-seq data, differing in their algorithmic approach. They can be categorized into four main classes: correlation-, regression-, mutual-information-, or modelling-based approaches.[4] In this study, we evaluated PIDC, GENIE3, and GRNBoost2, which have previously been recommended by Pratapa et al.[4] Moreover, we included PPCOR as a partial regression-based algorithm providing a baseline GRN algorithm. We use the imputed and unimputed scRNA-seq data as input matrices for network reconstruction with PIDC, GENIE3, and GRNBoost2 using default parameters. To this end, we use the evaluation framework BEELINE (version 1.0). In order to evaluate PPCOR results, we adjusted the code of the BEELINE framework. In the publicly available version of BEELINE, PPCOR considers the corrected p values of each reported interaction with its respective partial correlation value. However, in our case there were only NAs produced due to ill-conditioned matrices. Thus, we discard the p values and use a threshold of 0.1 absolute partial correlation value and selected interactions higher than the threshold.

As part of the BEELINE pipeline, we first run BLRunner.py to reconstruct the networks. Network reconstruction methods may compute undirected or directed edges, while the STRING database contains undirected edges. Thus, in evaluating a network reconstruction method that predicts undirected edges, for both STRING and predicted networks undirected edges get substituted by two opposing directed edges. For the comparison with the evaluation networks, we only consider and filter for edges going out of TFs. With this convention, bidirectional edges get counted only once (except where two TFs are connected by an interaction). This is meant to minimize the advantage that a method producing undirected edges might possibly have.

Finally, we use BLevaluater.py to compute early precision scores evaluating the performance of each network by comparing with to an evaluation network. Here, we choose the functional protein-protein interaction database STRING and cell-type-specific ChIP-seq -derived network provided by the BEELINE framework. We filter the network genes that only occur in the input expression matrix.

By using early precision scores, we only analyze the top-k networks.

Characterizing the reconstructed networks

Top-k network

For comparability reasons, we focus our analyses on the top-k networks. The top-k network of a reconstructed network includes the first k interactions selected by their ranks which were assigned by edge weights in descending order. Here, *k* represents the number of positive interactions in the evaluation network. Interactions can share the same ranks (e.g., the forward and backward interactions in an undirected graph). So with *k* interactions reported in the evaluation network, we select all interactions whose ranks are lower than or equal to *k*, obtaining the top-k network. Note that the number of reported interactions can be higher than *k*.

Network density and node degree

Taking into account the interaction between TFs and genes, only the network density is calculated by numEdges/((numGenes × numTFs) − numTFs).

In order to calculate the node degree, we consider all out- and incoming edges for a given node.

Methodology of evaluation

EPRs

Most of the network reconstruction algorithms infer networks that are close to a full graph. Being more interested in the most important predicted interactions, we analyzed the interactions with the highest (absolute) weights and thus the top ranked interactions within the network (top-k network). For this reason and to evaluate the performance of each inferred network based on using early precision (EP) scores, which is given by the number of TPs divided by the number of positively predicted observations within the top-k network. EP scores were calculated using BEELINE. Each dataset has a different underlying evaluation subnetwork and, hence, different evaluations regarding the random predictor. To account for these differences and in order to maintain comparability across datasets, we divide the EP scores by the network density (see formula above) of each evaluation subnetwork obtaining EPRs. Thus, EPR of 1 is indicative of a random predictor in all experimental datasets. To compare the performance of network inference in each imputation method with the corresponding unimputed data, we calculate log2 ratios between EPR imputed and EPR unimputed.

ANOVA

For each dataset, we performed a separate two-way ANOVA using the built-in R function. First, we fit a linear model using the log2 ratios of EPR values as a target variable and both the GRN algorithm and the imputation as regressor variables. Second, we summarize the variance model of the linear fit and report the p values. The source code is included on the github page.

Network similarities

In order to compare similarities across the reconstructed networks, we select the top 500 interactions reported in each model. Given two networks, similarity scores are obtained by the Jaccard index, which is defined as the number of overlapping interactions divided by the number of unified reported interactions. Repeating this in a pairwise iterative manner, we obtain a similarity matrix, which we use as an input for a heatmap that is clustered row- and column-wise (pheatmap R package).

We calculate ARI scores (mclust R package) in order to evaluate the clustering results based on an annotation label.[33] As annotation labels, we use the network reconstruction algorithm as well as the imputation method. We compare ARI scores across datasets obtained by the two labels using the pairwise Wilcoxon rank sum test.

SUPPLEMENTAL INFORMATION

Supplemental information can be found online at https://doi.org/10.1016/j.patter.2021.100414.

ACKNOWLEDGMENTS

We thank Mahsa Ghanbari and Prabhav Kalaghatgi for comments and proofreading our manuscript, Stefan Börno for assistance with the scRNA-seq processing, and Thomas Kreitler for technical support.

AUTHOR CONTRIBUTIONS

Conceptualization, L.H.L. and M.V.; methodology, L.H.L. and M.V.; software, formal analysis, and visualization, L.H.L.; writing – original draft, L.H.L. and M.V.; supervision, M.V.

DECLARATION OF INTERESTS

The authors declare no competing interests.

Received: July 12, 2021
Revised: July 30, 2021
Accepted: November 25, 2021
Published: December 22, 2021

REFERENCES

1. Aibar, S., González-Blas, C.B., Moerman, T., Huynh-Thu, V.A., Imrichova, H., Hulselmans, G., Rambow, F., Marine, J.-C., Geurts, P., Aerts, J., et al. (2017). SCENIC: single-cell regulatory network inference and clustering. Nat. Methods *14*, 1083–1086.
2. Chan, T.E., Stumpf, M.P.H., and Babtie, A.C. (2017). Gene regulatory network inference from single-cell data using multivariate information measures. Cell Syst *5*, 251–267.e3.
3. Ocone, A., Haghverdi, L., Mueller, N.S., and Theis, F.J. (2015). Reconstructing gene regulatory dynamics from high-dimensional single-cell snapshot data. Bioinformatics *31*, i89–96.
4. Pratapa, A., Jalihal, A.P., Law, J.N., Bharadwaj, A., and Murali, T.M. (2020). Benchmarking algorithms for gene regulatory network inference from single-cell transcriptomic data. Nat. Methods *17*, 147–154.
5. Qiu, X., Rahimzamani, A., Wang, L., Ren, B., Mao, Q., Durham, T., McFaline-Figueroa, J.L., Saunders, L., Trapnell, C., and Kannan, S. (2020). Inferring causal gene regulatory networks from coupled single-cell expression dynamics using scribe. Cell Syst *10*, 265–274.e11.
6. Specht, A.T., and Li, J. (2017). LEAP: constructing gene co-expression networks for single-cell RNA-sequencing data using pseudotime ordering. Bioinformatics *33*, 764–766.
7. Cha, J., and Lee, I. (2020). Single-cell network biology for resolving cellular heterogeneity in human diseases. Exp. Mol. Med. *52*, 1798–1808.
8. Iacono, G., Massoni-Badosa, R., and Heyn, H. (2019). Single-cell transcriptomics unveils gene regulatory network plasticity. Genome Biol. *20*, 110.
9. Luecken, M.D., and Theis, F.J. (2019). Current best practices in single-cell RNA-seq analysis: a tutorial. Mol. Syst. Biol. *15*, e8746.
10. Vieth, B., Parekh, S., Ziegenhain, C., Enard, W., and Hellmann, I. (2019). A systematic evaluation of single cell RNA-seq analysis pipelines. Nat. Commun. *10*, 4667.
11. Cole, M.B., Risso, D., Wagner, A., DeTomaso, D., Ngai, J., Purdom, E., Dudoit, S., and Yosef, N. (2019). Performance assessment and selection of normalization procedures for single-cell RNA-seq. Cell Syst *8*, 315–328.e8.
12. Lun, A.T.L., Bach, K., and Marioni, J.C. (2016). Pooling across cells to normalize single-cell RNA sequencing data with many zero counts. Genome Biol. *17*, 75.
13. Chen, M., and Zhou, X. (2018). VIPER: variability-preserving imputation for accurate gene expression recovery in single-cell RNA sequencing studies. Genome Biol. *19*, 196.
14. van Dijk, D., Sharma, R., Nainys, J., Yim, K., Kathail, P., Carr, A.J., Burdziak, C., Moon, K.R., Chaffer, C.L., Pattabiraman, D., et al. (2018). Recovering gene interactions from single-cell data using data diffusion. Cell *174*, 716–729.e27.
15. Eraslan, G., Simon, L.M., Mircea, M., Mueller, N.S., and Theis, F.J. (2019). Single-cell RNA-seq denoising using a deep count autoencoder. Nat. Commun. *10*, 390.
16. Huang, M., Wang, J., Torre, E., Dueck, H., Shaffer, S., Bonasio, R., Murray, J.I., Raj, A., Li, M., and Zhang, N.R. (2018). SAVER: gene expression recovery for single-cell RNA sequencing. Nat. Methods *15*, 539–542.
17. Lähnemann, D., Köster, J., Szczurek, E., McCarthy, D.J., Hicks, S.C., Robinson, M.D., Vallejos, C.A., Campbell, K.R., Beerenwinkel, N., Mahfouz, A., et al. (2020). Eleven grand challenges in single-cell data science. Genome Biol. *21*, 31.
18. Lopez, R., Regier, J., Cole, M.B., Jordan, M.I., and Yosef, N. (2018). Deep generative modeling for single-cell transcriptomics. Nat. Methods *15*, 1053–1058.
19. Tang, W., Bertaux, F., Thomas, P., Stefanelli, C., Saint, M., Marguerat, S., and Shahrezaei, V. (2020). bayNorm: Bayesian gene expression recovery, imputation and normalization for single-cell RNA-sequencing data. Bioinformatics *36*, 1174–1181.
20. Wagner, F., Yan, Y., and Yanai, I. (2017). K-nearest neighbor smoothing for high-throughput single-cell RNA-seq data. BioRxiv. https://doi.org/10.1101/217737.
21. Hou, W., Ji, Z., Ji, H., and Hicks, S.C. (2020). A systematic evaluation of single-cell RNA-sequencing imputation methods. Genome Biol. *21*, 218.
22. Blencowe, M., Arneson, D., Ding, J., Chen, Y.-W., Saleem, Z., and Yang, X. (2019). Network modeling of single-cell omics data: challenges, opportunities, and progresses. Emerg. Top. Life Sci. *3*, 379–398.
23. Breda, J., Zavolan, M., and van Nimwegen, E.J. (2019). Bayesian inference of the gene expression states of single cells from scRNA-seq data. BioRxiv. https://doi.org/10.1101/2019.12.28.889956.
24. Chu, L.-F., Leng, N., Zhang, J., Hou, Z., Mamott, D., Vereide, D.T., Choi, J., Kendziorski, C., Stewart, R., and Thomson, J.A. (2016). Single-cell RNA-seq reveals novel regulators of human embryonic stem cell differentiation to definitive endoderm. Genome Biol. *17*, 173.
25. Camp, J.G., Sekine, K., Gerber, T., Loeffler-Wirth, H., Binder, H., Gac, M., Kanton, S., Kageyama, J., Damm, G., Seehofer, D., et al. (2017). Multilineage communication regulates human liver bud development from pluripotency. Nature *546*, 533–538.
26. Hayashi, T., Ozaki, H., Sasagawa, Y., Umeda, M., Danno, H., and Nikaido, I. (2018). Single-cell full-length total RNA sequencing uncovers dynamics of recursive splicing and enhancer RNAs. Nat. Commun. *9*, 619.
27. Shalek, A.K., Satija, R., Shuga, J., Trombetta, J.J., Gennert, D., Lu, D., Chen, P., Gertner, R.S., Gaublomme, J.T., Yosef, N., et al. (2014). Single-cell RNA-seq reveals dynamic paracrine control of cellular variation. Nature *510*, 363–369.
28. Nestorowa, S., Hamey, F.K., Pijuan Sala, B., Diamanti, E., Shepherd, M., Laurenti, E., Wilson, N.K., Kent, D.G., and Göttgens, B. (2016). A single-cell resolution map of mouse hematopoietic stem and progenitor cell differentiation. Blood *128*, e20–31.
29. Huynh-Thu, V.A., Irrthum, A., Wehenkel, L., and Geurts, P. (2010). Inferring regulatory networks from expression data using tree-based methods. PLoS ONE *5*, e12776.
30. Moerman, T., Aibar Santos, S., Bravo González-Blas, C., Simm, J., Moreau, Y., Aerts, J., and Aerts, S. (2019). GRNBoost2 and Arboreto: efficient and scalable inference of gene regulatory networks. Bioinformatics *35*, 2159–2161.
31. Kim, S. (2015). ppcor: an R package for a fast calculation to semi-partial correlation coefficients. Commun. Stat. Appl. Methods *22*, 665–674.

CellPress
OPEN ACCESS

32. Szklarczyk, D., Gable, A.L., Lyon, D., Junge, A., Wyder, S., Huerta-Cepas, J., Simonovic, M., Doncheva, N.T., Morris, J.H., Bork, P., et al. (2019). STRING v11: protein-protein association networks with increased coverage, supporting functional discovery in genome-wide experimental datasets. Nucleic Acids Res. *47*, D607–D613.

33. Hubert, L., and Arabie, P. (1985). Comparing partitions. J. Classification *2*, 193–218.

34. Gates, A.J., and Ahn, Y.-Y. (2017). The impact of random models on clustering similarity. BioRxiv. https://doi.org/10.1101/196840.

35. Ghanbari, M., Lasserre, J., and Vingron, M. (2019). The distance precision matrix: computing networks from non-linear relationships. Bioinformatics *35*, 1009–1017.

36. Chen, S., and Mar, J.C. (2018). Evaluating methods of inferring gene regulatory networks highlights their lack of performance for single cell gene expression data. BMC Bioinformatics *19*, 232.

37. Steinheuer, L.M., Canzler, S., and Hackermüller, J. (2021). Benchmarking scRNA-seq imputation tools with respect to network inference highlights deficits in performance at high levels of sparsity. BioRxiv. https://doi.org/10.1101/2021.04.02.438193v1.

38. Andrews, T.S., and Hemberg, M. (2018). False signals induced by single-cell imputation, version 2; peer review: 4 approved. F1000Res *7*, 1740.

39. Dobin, A., Davis, C.A., Schlesinger, F., Drenkow, J., Zaleski, C., Jha, S., Batut, P., Chaisson, M., and Gingeras, T.R. (2013). STAR: ultrafast universal RNA-seq aligner. Bioinformatics *29*, 15–21.

Patterns, Volume *3*

Supplemental information

Effect of imputation on gene network reconstruction from single-cell RNA-seq data

Lam-Ha Ly and Martin Vingron

Supplemental Information

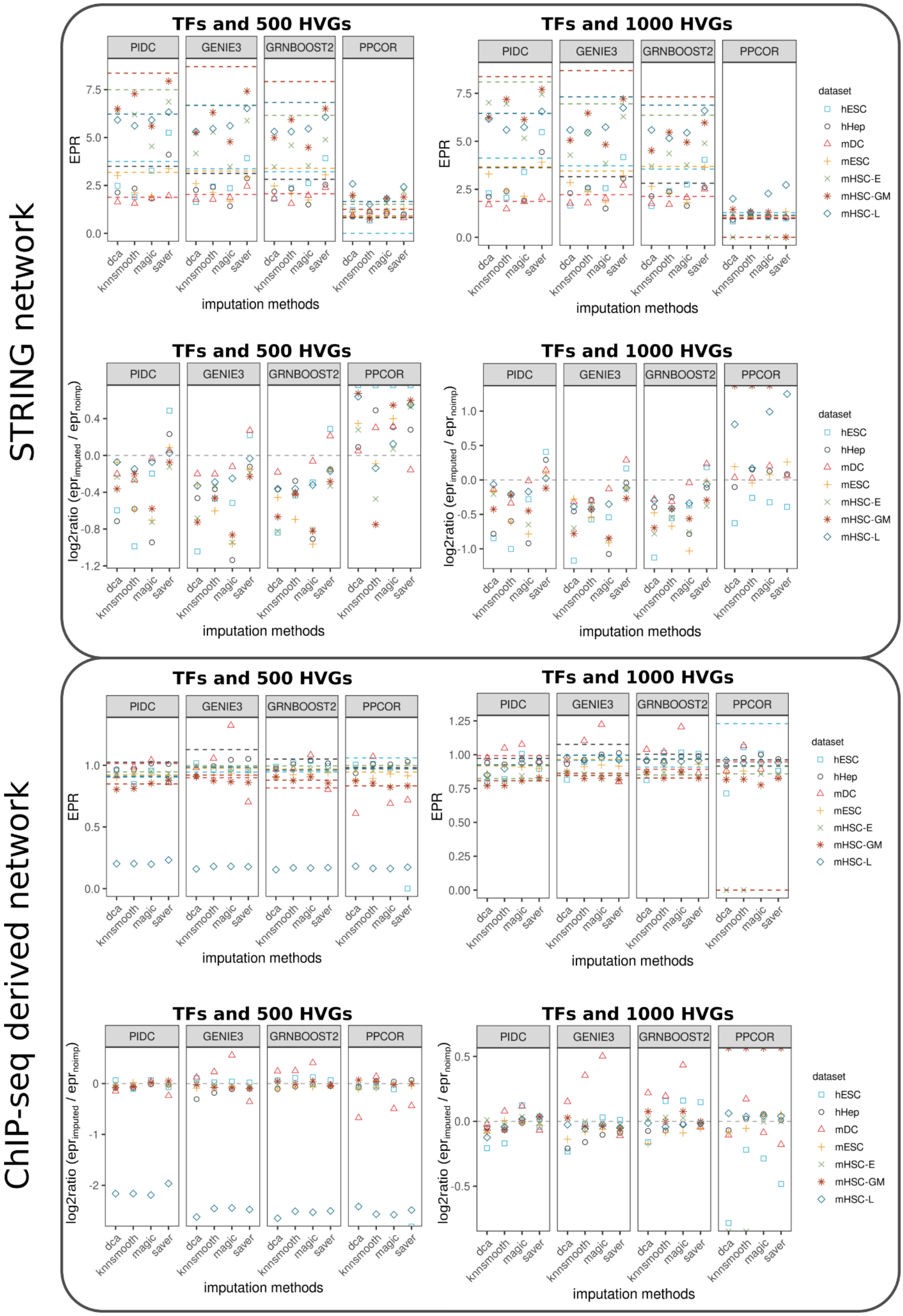

Fig. S1 | Performance scores of network models including PPCOR compared to STRING and ChIP-seq derived networks as evaluation networks. Upper panel is related to to Fig. 2 comparing

the inferred network results with the STRING network. Here, we include PPCOR as a baseline GRN algorithm. PPCOR performs almost similarly to a random predictor. In some cases PPCOR failed to run due to ill-conditioned data matrices corresponding to EPR scores equal to 0. Below panel compares the inferred network results with cell type-specific ChIP-seq derived networks. In both prefiltered datasets the performances are close to random. Imputation does not improve the network predictions. Due to normalization by using the network density, the EPR scores in mHSC-L imputed data differ strongly from the unimputed data. Here, low numbers of genes/ TFs and edges lead to different network densities (see Data S1). In both evaluation scenarios the results between the prefiltered datasets based on the number of highly variable genes (HVGs) are comparable. Hence, varying the number of genes has little effect on the network performance predictions.

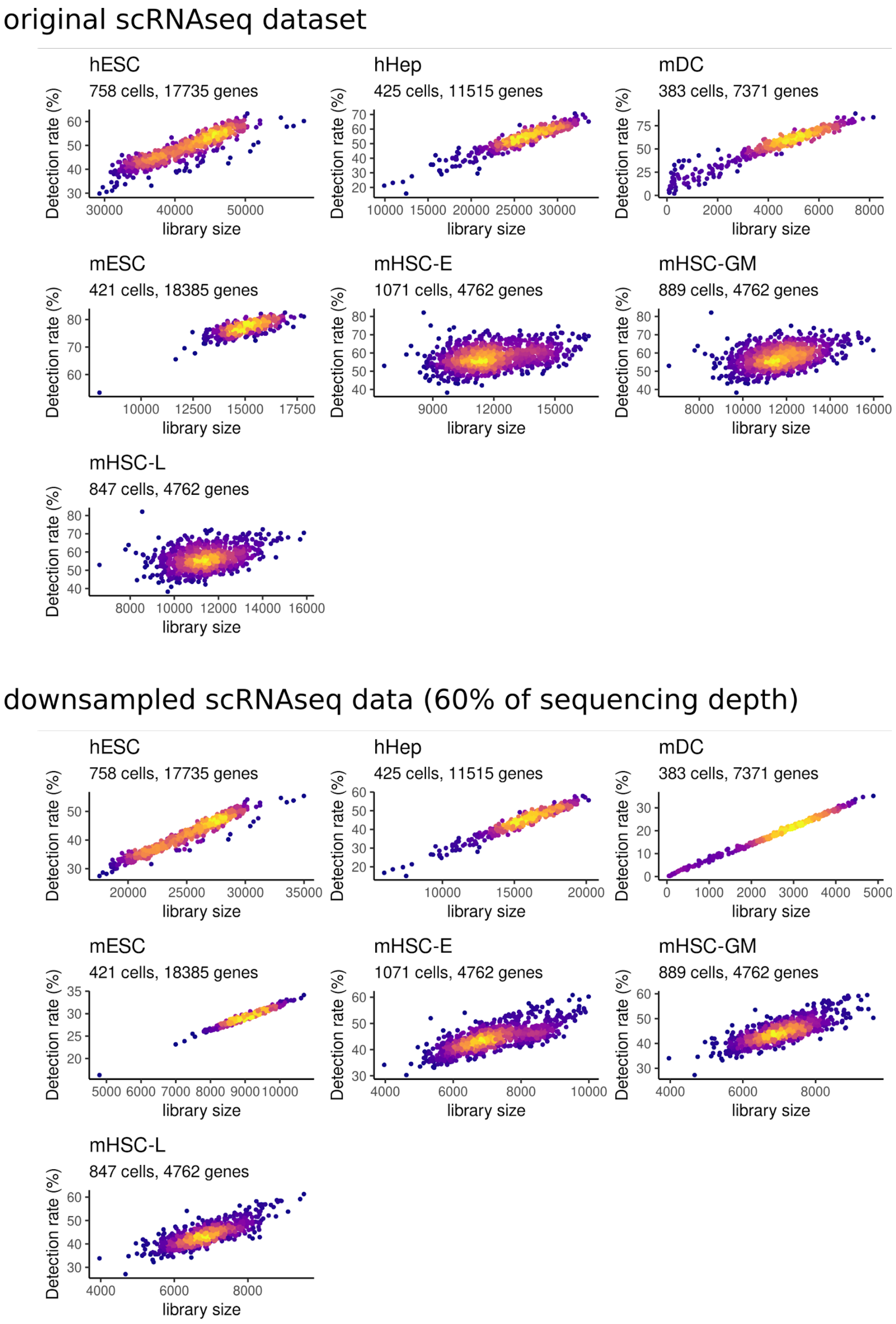

Fig. S2 | Gene detection rate and library size in experimental scRNAseq datasets (original and downsampled). Scatterplots colored by density of points (cells). Gene detection using a threshold of gene count > 0. Library size determined by the sum of all gene counts. Downsampling procedure

performed by sampling n times (60% of the original library size) according to the multinomial distribution.

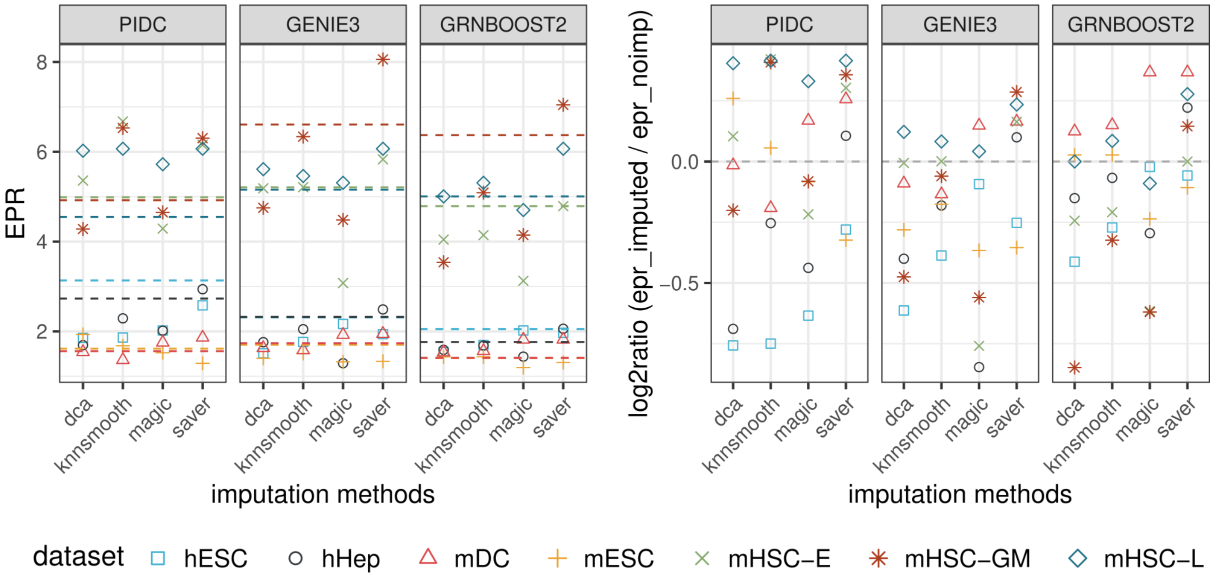

Fig. S3 | Performance measures of network models obtained by downsampled dataset. Performance scores reported on downsampled scRNAseq data (60% of original library size) and prefiltered dataset (TFs and 500 HVGs). (Left) Absolute EPR scores. Dashed line represents EPR scores obtained without imputation. (Right) log2-ratios between imputed and unimputed EPR scores. Log2ratio = 0 represents no change in performance (grey dashed line) after imputation. Generally, more improvements (positive log2ratios) than in the respective column of Figure 2 (TFs and 500 HVGs).

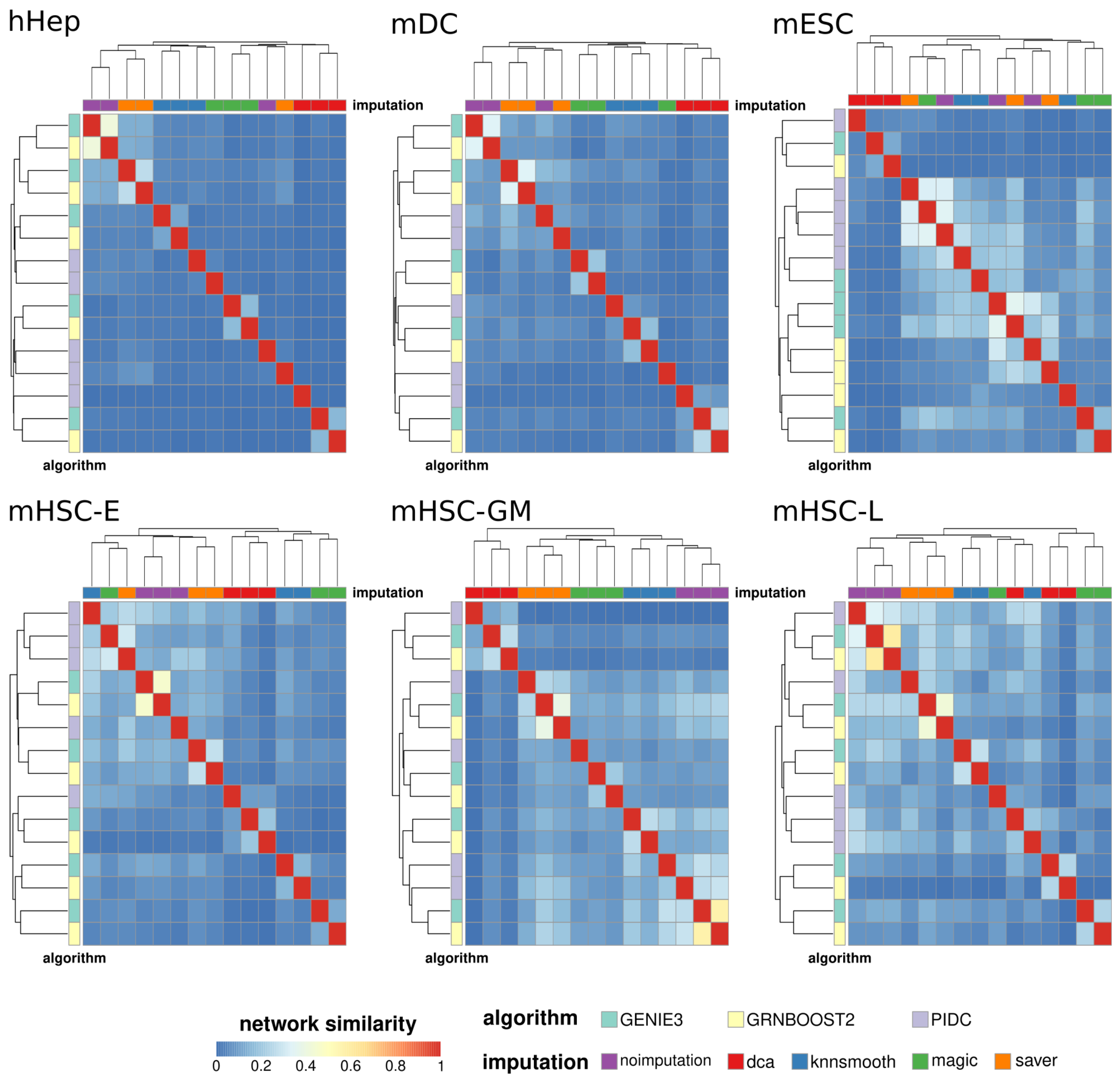

Fig. S4 | Network similarities across all models and cell types. Related to Figure 3B we inspect the heatmap of network similarities of the remaining cell types. Network similarity scores obtained by pairwise Jaccard index from top500 interactions. Columns are annotated by imputation method, rows are annotated by GRN reconstruction algorithm.

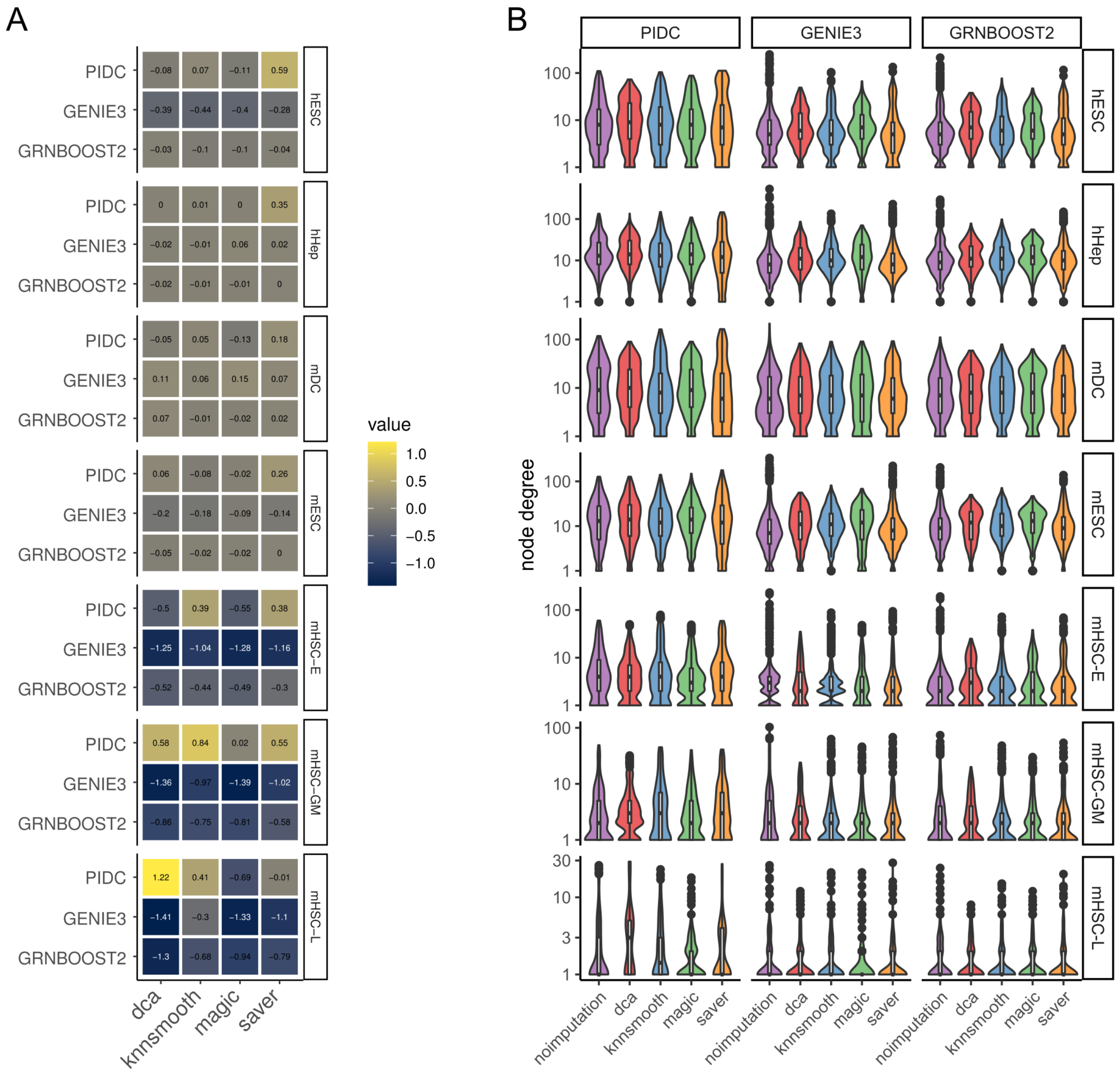

Fig. S5 | Structural changes in inferred networks. (A) Change of node density before and after imputation. Log2 ratios between density (after imputation) and density (before imputation) are color-coded. Positive values represent a denser whereas negative values represent a more sparse network with respect to the unimputed model. (B) Node degree distribution across all models. Y-axis is log-scaled.

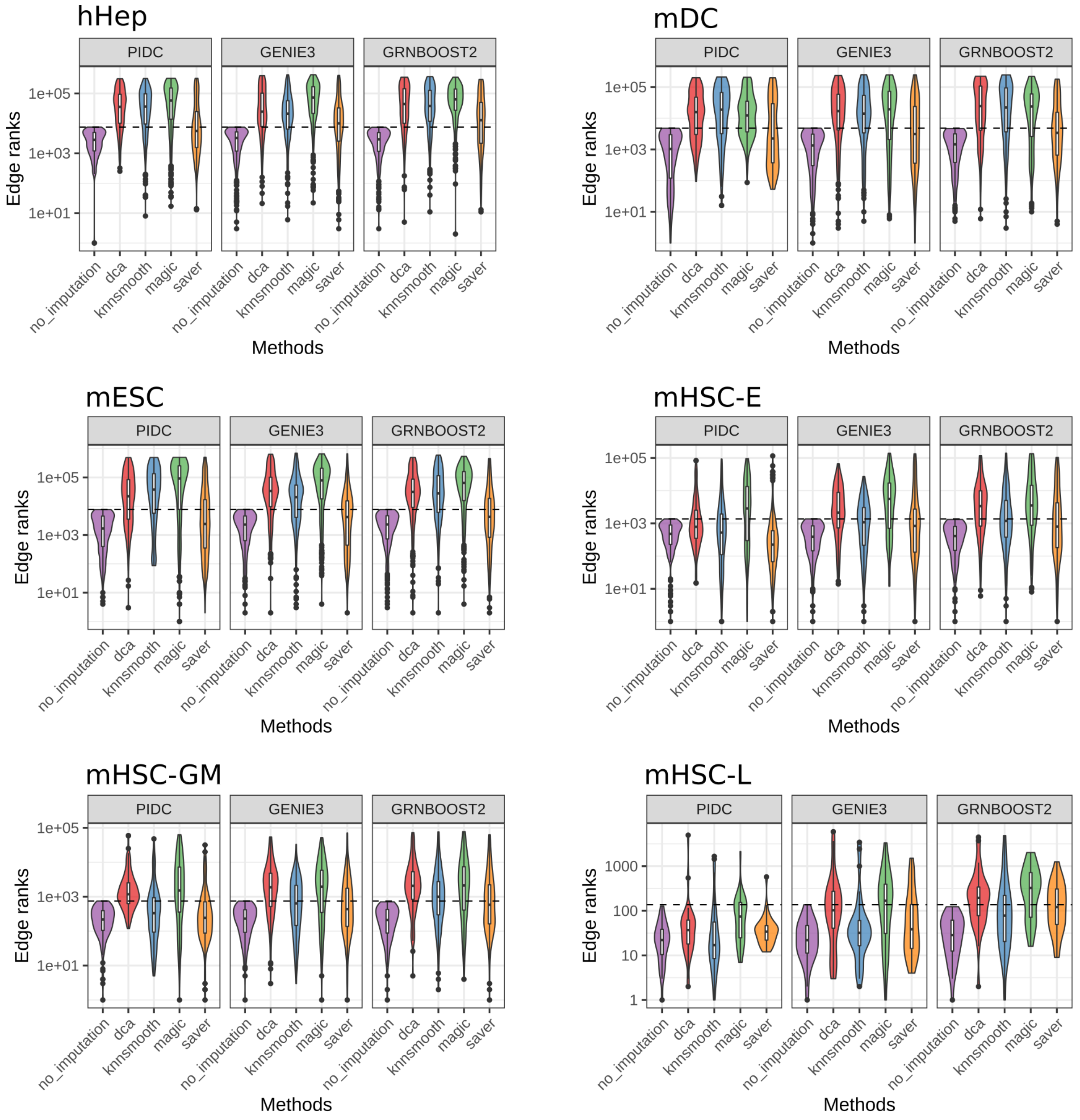

Fig. S6: True positive interactions identified on unimputed data and their change in edge ranks after imputation. Related to Figure 4C we inspect the change of unimputed TP ranks after imputation in the remaining cell types. Corrected p-values obtained by Wilcoxon rank sum test can be taken from Tab. S2.

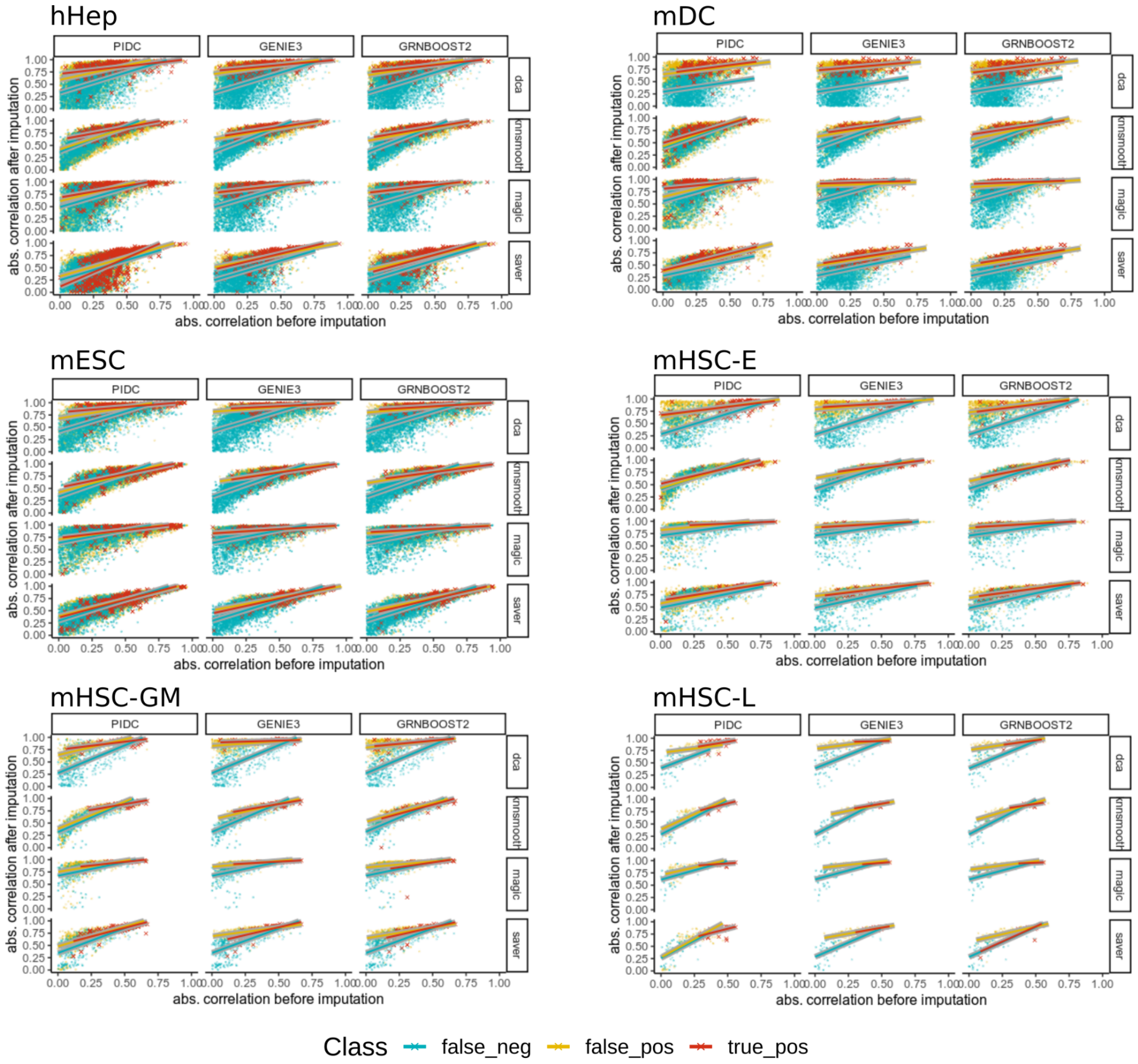

Fig. S7: Absolute Pearson's correlation coefficients before and after imputation colored by prediction class obtained in each model. Related to Figure 4D we inspect the change of correlation values for TP, FP and FN classified by each model in the remaining cell types. Colors correspond to the prediction classes in each model.

data	factor	sum of squares	mean of squares	p-value
hESC	GRN	0.0455	0.0228	0.68436
	imputation	2.3036	0.7679	0.00435 **
hHep	GRN	0.0205	0.0103	0.75904
	imputation	1.4728	0.4909	0.00421 **
mDC	GRN	0.0061	0.00307	0.68601
	imputation	0.3728	0.12428	0.00275 **
mESC	GRN	0.1324	0.0662	0.00638 **
	imputation	1.1478	0.3826	3.64e-05 ***
mHSC-E	GRN	0.1456	0.07281	0.07206 .
	imputation	0.6497	0.21657	0.00541 **
mHSC-GM	GRN	0.1748	0.08739	0.000247 ***
	imputation	0.5445	0.18151	2.02e-05 ***
mHSC-L	GRN	0.11682	0.05841	0.000572 ***
	imputation	0.08218	0.02739	0.003099 **

Tab. S1 | Analysis of variance of performance scores for each dataset. ANOVA results on EPR log-fold-ratios. Significance codes are 0 '***' 0.001 '**' 0.01 '*' 0.05 '.' 0.1 ' ' 0. Higher significance p-values in imputation give evidence that a higher variance within imputation methods compared to GRN algorithms is prevalent, and vice versa.

data	imputation	PIDC	GENIE3	GRNBOOST2
hESC	dca	6.23E-59	1.35E-52	3.22E-57
	knnsmooth	9.30E-70	5.56E-30	1.72E-35
	magic	5.41E-45	1.89E-47	1.68E-51
	saver	1.99E-15	5.46E-12	8.93E-13
hHep	dca	2.77E-144	3.67E-89	5.47E-83
	knnsmooth	1.11E-130	3.88E-85	5.87E-86
	magic	1.26E-126	1.19E-125	1.86E-108
	saver	1.66E-23	1.08E-37	1.34E-29
mDC	dca	1.15E-48	1.91E-39	8.93E-42
	knnsmooth	1.47E-46	5.11E-38	3.20E-38
	magic	2.14E-58	6.35E-31	9.12E-32
	saver	2.69E-10	7.78E-08	3.66E-10
mESC	dca	2.85E-75	6.53E-79	7.09E-83
	knnsmooth	2.84E-75	9.74E-59	5.33E-77
	magic	1.54E-78	1.25E-80	4.75E-90
	saver	3.61E-05	3.28E-07	7.37E-10
mHSC-E	dca	1.93E-08	2.97E-24	6.55E-25
	knnsmooth	1	4.70E-07	1.72E-10
	magic	2.44E-14	6.80E-27	4.39E-26
	saver	1	0.001687	0.0004226
mHSC-GM	dca	5.32E-44	1.02E-33	7.75E-32
	knnsmooth	0.05846	2.32E-09	9.27E-17
	magic	8.66E-21	2.31E-23	2.89E-22
	saver	1	8.45E-07	6.18E-09
mHSC-L	dca	1	0.006637	1.51E-05
	knnsmooth	1	1	0.3427

	magic	0.005388	0.004132	3.54E-06
	saver	0.7522	1	0.0006845

Tab. S2: Statistical testing of differences between edge rank distributions. Corrected p-values (Bonferroni method) obtained after Wilcoxon rank sum test between ranks of unimputed true positive edges and their respective ranks after imputation (see Fig. 4C, Fig. S6).
